# Supplementary figures and images for: Genomic prediction of rice mesocotyl length indicative of directing seeding suitability using a half-sib hybrid population
Source: PLoS One. 2023 Apr 5;18(4):e0283989. doi: 10.1371/journal.pone.0283989 (PMC10075464; doi:10.1371/journal.pone.0283989)

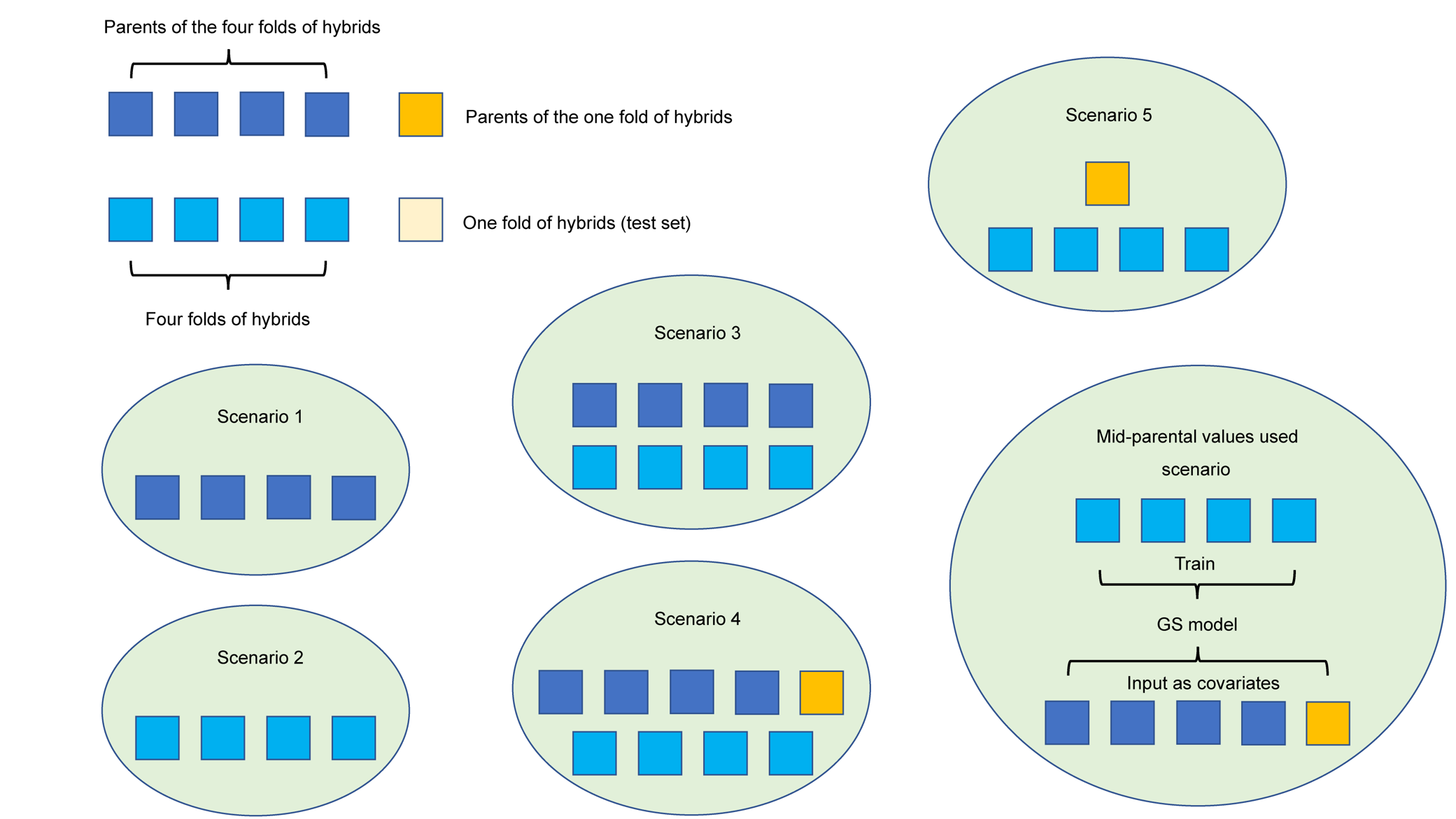

Supplement: S1 Fig — (TIF) [file pone.0283989.s001.tif]

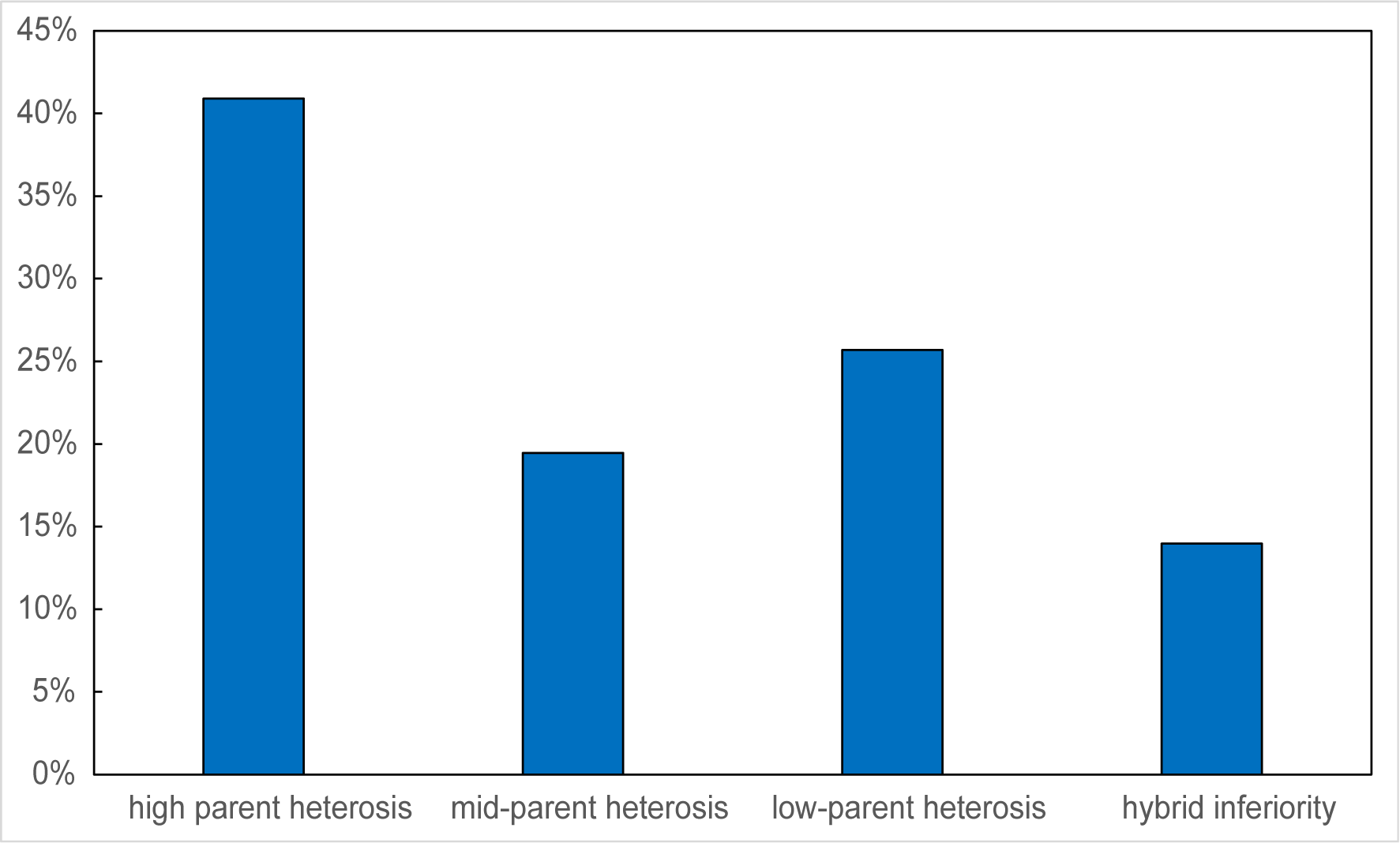

Supplement: S2 Fig — (TIF) [file pone.0283989.s002.tif]
