# Supplementary material for: Genomic prediction of rice mesocotyl length indicative of directing seeding suitability using a half-sib hybrid population
Source: PLoS One. 2023 Apr 5;18(4):e0283989. doi: 10.1371/journal.pone.0283989 (PMC10075464; doi:10.1371/journal.pone.0283989)
Supplement: S1 Table — (DOCX) [file pone.0283989.s003.docx]

**Supplementary Table S1.** The information of 402 rice accessions.

| Accession ID | Accession name |
| --- | --- |
| 1 | Taifeng A |
| 2 | BHORO NATA::IRGC 31728 / IR 36 |
| 3 | BHUA BALAM::IRGC 31729 / IR 36 |
| 4 | CHAP SAIL::IRGC 31755 / IR 36 |
| 5 | CHING JHORA::IRGC 31761 / IR 36 |
| 6 | BAZAIL 1187::IRGC 32819 / IR 36 |
| 7 | CHOTTO MOLLIK::IRGC 34687 / IR 36 |
| 8 | ASHPALI::IRGC 36980 / IR 36 |
| 9 | BHASAMANIK::IRGC 37014 / IR 36 |
| 10 | BOALERDAT::IRGC 37020 / IR 36 |
| 11 | BLACK 28-577::IRGC 37828 / IR 36 |
| 12 | BLACK 28-578::IRGC 37829 / IR 36 |
| 13 | BLACK 28-579::IRGC 37830 / IR 36 |
| 14 | BR 52-87-1::IRGC 39191 / IR 36 |
| 15 | CHOTO BAWALIA::IRGC 49268 / IR 36 |
| 16 | BADI::IRGC 49300 / IR 36 |
| 17 | BALA DHAL KUSRI::IRGC 49301 / IR 36 |
| 18 | BARA LONA KURCHI::IRGC 49302 / IR 36 |
| 19 | BATHAG::IRGC 49304 / IR 36 |
| 20 | BHAHURI (AWN)::IRGC 49308 / IR 36 |
| 21 | CASH::IRGC 49310 / IR 36 |
| 22 | CHANGAI::IRGC 49311 / IR 36 |
| 23 | BOROCHINAL::IRGC 133882 / IR 36 |
| 24 | BHADOIA 689::IRGC 6536 / IR 36 |
| 25 | APCHAYA::IRGC 26535 / IR 36 |
| 26 | CHIKON::IRGC 31757 / IR 36 |
| 27 | CHINRAVUCHI::IRGC 37048 / IR 36 |
| 28 | AWNED 25-546::IRGC 37782 / IR 36 |
| 29 | AWNED 25-550::IRGC 37786 / IR 36 |
| 30 | BLACK 28-557::IRGC 37808 / IR 36 |
| 31 | BLACK 28-571::IRGC 37822 / IR 36 |
| 32 | BLACK 28-573::IRGC 37824 / IR 36 |
| 33 | BHABANI (JABAIN)::IRGC 49266 / IR 36 |
| 34 | CHAULA MAGHI::IRGC 49312 / IR 36 |
| 35 | BR 10::IRGC 53457 / IR 36 |
| 36 | BR 11::IRGC 53458 / IR 36 |
| 37 | BARAROPA::IRGC 53470 / IR 36 |
| 38 | AMAIRRA::IRGC 79305 / IR 36 |
| 39 | BOROHAJI::IRGC 114597 / IR 36 |
| 40 | BARAN BORO::IRGC 117434 / IR 36 |
| 41 | CHANDARHAT::IRGC 121605 / IR 36 |
| 42 | BAILA BORKI::IRGC 133693 / IR 36 |
| 43 | BASFUL 714::IRGC 133714 / IR 36 |
| 44 | MICOCHU::IRGC 121055 / IR 36 |
| 45 | CHAPLAISH::IRGC 25846 / IR 36 |
| 46 | DV 123::IRGC 117724 / IR 36 |
| 47 | DJ 123::IRGC 117711 / IR 36 |
| 48 | HOLOI BASH (SOLOI BASH)::IRGC 120969 / IR 36 |
| 49 | TCHAMPA::IRGC 117585 / IR 36 |
| 50 | JAMBALI BUSSA::IRGC 120995 / IR 36 |
| 51 | KANGRI::IRGC 127488 / IR 36 |
| 52 | RANRUWAN::IRGC 127763 / IR 36 |
| 53 | KALAR KAR::IRGC 49737 / IR 36 |
| 54 | TAK:IRGC 121582 / IR 36 |
| 55 | KARENDOL::IRGC 37485 / IR 36 |
| 56 | BOILAN:::IRGC 120899 / IR 36 |
| 57 | DV 60::IRGC 8832 / IR 36 |
| 58 | NATEL BORO::IRGC 127652 / IR 36 |
| 59 | DJ 69::IRGC 127324 / IR 36 |
| 60 | INDIA DULAR (NO ORDEM 7)::IRGC 127430 / IR 36 |
| 61 | KOALARETA::IRGC 121624 / IR 36 |
| 62 | ARC 11959::IRGC 121188 / IR 36 |
| 63 | M 136-20::IRGC 35053 / IR 36 |
| 64 | DA 27-C::IRGC 45628 / IR 36 |
| 65 | T 65::IRGC 52773 / IR 36 |
| 66 | NAYIMA::IRGC 121447 / IR 36 |
| 67 | JC 148::IRGC 9069 / IR 36 |
| 68 | ARC 7336::IRGC 127169 / IR 36 |
| 69 | UPRH 58::IRGC 127879 / IR 36 |
| 70 | GHAIYA::IRGC 128297 / IR 36 |
| 71 | CHAMKA::IRGC 25844 / IR 36 |
| 72 | BENA JHUPI::IRGC 127993 / IR 36 |
| 73 | BLACK 28-576::IRGC 37827 / IR 36 |
| 74 | BINNI DHAN::IRGC 26569 / IR 36 |
| 75 | CHENGA SAIL::IRGC 31756 / IR 36 |
| 76 | BRJ 1-13 B-55::IRGC 32582 / IR 36 |
| 77 | AWNED 25-545::IRGC 37781 / IR 36 |
| 78 | BLACK 28-553::IRGC 37804 / IR 36 |
| 79 | BOW PAGAL::IRGC 43796 / IR 36 |
| 80 | BR 194-1-2-1-2::IRGC 44013 / IR 36 |
| 81 | BATA::IRGC 53473 / IR 36 |
| 82 | AGUNI KARTIKSAIL::IRGC 77213 / IR 36 |
| 83 | TA LAY |
| 84 | HASAWI |
| 85 | POKKALI 4 |
| 86 | CHERIVIRUPPU |
| 87 | GETU |
| 88 | AKUNDI |
| 89 | TAL MUGUR |
| 90 | HASSAN TAREME |
| 91 | BPI RI 2 |
| 92 | ARC 18567 |
| 93 | ORUMUNDAKAN |
| 94 | WALIMBO |
| 95 | RD 23 |
| 96 | MSALIM JARO |
| 97 | LAN SHENG |
| 98 | IR 8866-30-3-1-4-2 |
| 99 | CSR 11 |
| 100 | PSBRC 50 |
| 101 | POKKALI |
| 102 | NSIC RC 106 |
| 103 | IR 66946-3R-178-1-1 |
| 104 | FL 478 |
| 105 | IR 45427-2B-2-2B-1-1::G1 |
| 106 | CSR 28 |
| 107 | TCP-266-2-49-B-B-3 |
| 108 | BRRI DHAN 53 |
| 109 | POKKALI (8558) |
| 110 | AGIR JAIL BIROI::IRGC 66763-1 |
| 111 | CHAMKA |
| 112 | AUS 362 |
| 113 | IARI 5824::IRGC 14420 |
| 114 | KH 998::IRGC 16948 |
| 115 | DJ 99::IRGC 8465 |
| 116 | DJ 68::IRGC 8833 |
| 117 | DHULE BIZ::IRGC 31769 |
| 118 | DULPI::IRGC 31779 |
| 119 | DAINOS::IRGC 37053 |
| 120 | DEPA::IRGC 37056 |
| 121 | DUKSAIL::IRGC 37069 |
| 122 | DULOBECH::IRGC 37071 |
| 123 | ADT 8::IRGC 5921 |
| 124 | JC 70::IRGC 9114 |
| 125 | PATTI DHAN::IRGC 10019 |
| 126 | TENGREY::IRGC 10186 |
| 127 | KHURKIJOHA::IRGC 10187 |
| 128 | TENGARI LOCAL::IRGC 10188 |
| 129 | KRISHNA (CR 1-6)::IRGC 12889 |
| 130 | RATNA (CR 44-11)::IRGC 12890 |
| 131 | ASWATHI::IRGC 14783 |
| 132 | CR 57-29::IRGC 15775 |
| 133 | CR 60-10::IRGC 15777 |
| 134 | KH 864::IRGC 16949 |
| 135 | DA 5::IRGC 5855 |
| 136 | GANGA SAGAR::IRGC 31650 |
| 137 | CYLINDRICAL 30-662::IRGC 37900 |
| 138 | DA 31-1-1::IRGC 37901 |
| 139 | GANDI::IRGC 49186 |
| 140 | BHASMANIK::IRGC 624 |
| 141 | SR 26::IRGC 4918 |
| 142 | ASGO::IRGC 6657 |
| 143 | MAINAGURI::IRGC 10182 |
| 144 | BONGABAR::IRGC 10184 |
| 145 | IARI 10560::IRGC 14426 |
| 146 | KI 63-4::IRGC 34767 |
| 147 | KENDEL::IRGC 34991 |
| 148 | SXC 199::IRGC 35173 |
| 149 | TR 17::IRGC 36743 |
| 150 | TELLA HAMSA::IRGC 39541 |
| 151 | CRM 8-5708-3::IRGC 39583 |
| 152 | OR 87-9::IRGC 39672 |
| 153 | OR 117-22::IRGC 39682 |
| 154 | CNM 17::IRGC 45378 |
| 155 | GANGAJALIGHAT::IRGC 45674 |
| 156 | JS 52-67::IRGC 45945 |
| 157 | KAJALBHOG::IRGC 45951 |
| 158 | KANTHAMALA::IRGC 46044 |
| 159 | CO 11::IRGC 26841 |
| 160 | MTU 8002::IRGC 28516 |
| 161 | KI 68::IRGC 34768 |
| 162 | BHADRA::IRGC 34858 |
| 163 | TR 21::IRGC 36747 |
| 164 | TR 25::IRGC 36750 |
| 165 | CRHP 8::IRGC 36839 |
| 166 | BM 45::IRGC 36840 |
| 167 | AICRIP 111-17 (HPU 2181)::IRGC 39154 |
| 168 | CN 539::IRGC 39234 |
| 169 | IET 2300::IRGC 39268 |
| 170 | IET 3262::IRGC 39275 |
| 171 | KH 17854::IRGC 39476 |
| 172 | P 4-1-11-21::IRGC 39524 |
| 173 | TNAU 13471::IRGC 39542 |
| 174 | BPT 1235::IRGC 39575 |
| 175 | CNM 25::IRGC 39579 |
| 176 | CN 44-33-3::IRGC 39581 |
| 177 | CRK 30-40::IRGC 39582 |
| 178 | CRM 8-5710-8::IRGC 39585 |
| 179 | CRM 8-5712::IRGC 39586 |
| 180 | CR 129-65::IRGC 39596 |
| 181 | CR 189-62-12::IRGC 39618 |
| 182 | HG 60-49::IRGC 39636 |
| 183 | KRC 4::IRGC 39651 |
| 184 | OR 83-26::IRGC 39671 |
| 185 | OR 117-31::IRGC 39684 |
| 186 | PAU 21-88-5::IRGC 39691 |
| 187 | PAU 41-281-1-1::IRGC 39696 |
| 188 | PAU 41-306-2-1::IRGC 39697 |
| 189 | PAU 125-149-2::IRGC 39698 |
| 190 | PUSA 33-30-18-3::IRGC 39707 |
| 191 | RNR 56165-1::IRGC 39716 |
| 192 | RP 825-28-7-1::IRGC 39765 |
| 193 | RP 894-15-2-1-1::IRGC 39770 |
| 194 | RP 894-61-1-3-7-2::IRGC 39771 |
| 195 | RP 919-8-9-2-6-3::IRGC 39775 |
| 196 | RP 932-4-11-3-4::IRGC 39777 |
| 197 | RP 967-4-7-2-1::IRGC 39783 |
| 198 | RP 967-4-7-2-7::IRGC 39785 |
| 199 | RP 967-11-1-3-6::IRGC 39791 |
| 200 | RP 974-133-7-29-16-9-4::IRGC 39814 |
| 201 | RP 975-32-1-1-2::IRGC 39823 |
| 202 | SS 55-304::IRGC 39854 |
| 203 | TNAU 7583::IRGC 39860 |
| 204 | TNAU 9485-7::IRGC 39864 |
| 205 | OR 8023::IRGC 40016 |
| 206 | UPR 96-1::IRGC 40065 |
| 207 | RP 1303-80-1::IRGC 40141 |
| 208 | BLACK GORA S N 32::IRGC 44819 |
| 209 | BROWN GORA S N 12::IRGC 44832 |
| 210 | BROWN GORA S N 68::IRGC 44852 |
| 211 | AKANDARANGI::IRGC 44920 |
| 212 | BARAHARIN::IRGC 45083 |
| 213 | CHACHI::IRGC 45256 |
| 214 | CR 115-76::IRGC 45413 |
| 215 | DHIPISAIL::IRGC 45541 |
| 216 | GODABELKI::IRGC 45696 |
| 217 | HALDIJAB::IRGC 45746 |
| 218 | HALDIJAM::IRGC 45747 |
| 219 | HEERAMOTI::IRGC 45790 |
| 220 | HELFULPI::IRGC 45792 |
| 221 | IET 2233::IRGC 45839 |
| 222 | KABRANONA::IRGC 45947 |
| 223 | KALA RATA 1-24::IRGC 26913 |
| 224 | CUL 688::IRGC 28559 |
| 225 | TNAU 633::IRGC 28572 |
| 226 | BR 7::IRGC 33995 |
| 227 | DHUP SAIL::IRGC 34902 |
| 228 | NARARI SABRI::IRGC 35060 |
| 229 | K 140-52-3::IRGC 39511 |
| 230 | P 33-C-30::IRGC 39526 |
| 231 | CR 189-62-15::IRGC 39620 |
| 232 | CUL 3/RATNA 55::IRGC 39627 |
| 233 | PAU 41-10-1-3::IRGC 39694 |
| 234 | RP 189-3::IRGC 39740 |
| 235 | RP 825-92-4-16::IRGC 39767 |
| 236 | RP 967-65-4-3-7::IRGC 39802 |
| 237 | TNAU 13613::IRGC 39865 |
| 238 | VADAGAON 416-34-2-6::IRGC 39870 |
| 239 | BLACK GORA S N 19::IRGC 44818 |
| 240 | CR 148-2623-215::IRGC 45437 |
| 241 | CAROLINA::IRGC 3401 |
| 242 | MUSHKAN 41::IRGC 6418 |
| 243 | BELLO::IRGC 6658 |
| 244 | DZ 60::IRGC 8558 |
| 245 | SADRI::IRGC 33946 |
| 246 | GUNDIL::IRGC 34544 |
| 247 | KORGUT::IRGC 35003 |
| 248 | MAROANTRANO::IRGC 97278 |
| 249 | CYPRESS::IRGC 124359 |
| 250 | MOROBEREKAN::IRGC 117272 |
| 251 | BONDYL::IRGC 25842 |
| 252 | CHAKKAL::IRGC 25843 |
| 253 | AMBORO 1::IRGC 36970 |
| 254 | BASIRAJ::IRGC 37003 |
| 255 | BOTA::IRGC 37394 |
| 256 | CHOTA BAZAL::IRGC 37407 |
| 257 | ASAIL::IRGC 49052 |
| 258 | BHOLANATH::IRGC 64767 |
| 259 | BALAM 1::IRGC 77217 |
| 260 | BOLONGA::IRGC 77224 |
| 261 | BINI (BLACK)::IRGC 82142 |
| 262 | BOILAM::IRGC 87170 |
| 263 | CHINA IRRI::IRGC 87172 |
| 264 | BENTOBALA::IRGC 26948 |
| 265 | BAGIAMON 349::IRGC 6494 |
| 266 | BAKOI::IRGC 25836 |
| 267 | BASHPOR::IRGC 37002 |
| 268 | BENA GACHYA::IRGC 37380 |
| 269 | BUTUBALAM::IRGC 49165 |
| 270 | CHAPLAIS::IRGC 49168 |
| 271 | BADAI::IRGC 53464 |
| 272 | BODESHI::IRGC 53481 |
| 273 | BOITI::IRGC 53482 |
| 274 | ASHA::IRGC 66765 |
| 275 | BAMURA::IRGC 66770 |
| 276 | BIR MAZLA::IRGC 66771 |
| 277 | BHORIA AUS::IRGC 66830 |
| 278 | BALAM 2::IRGC 77218 |
| 279 | BHABANI::IRGC 77222 |
| 280 | BAIGUN GATI::IRGC 79308 |
| 281 | BARI BHADAR::IRGC 79309 |
| 282 | BOUMAL::IRGC 79320 |
| 283 | BINI (WHITE)::IRGC 82829 |
| 284 | AGUNBAN::IRGC 25828 |
| 285 | ARAI::IRGC 25830 |
| 286 | BHOULAM::IRGC 37244 |
| 287 | BADALI::IRGC 37362 |
| 288 | BUSHRI::IRGC 37396 |
| 289 | CHALAKI::IRGC 37398 |
| 290 | ASSHINI::IRGC 53461 |
| 291 | 三七早 |
| 292 | 广场矮6号 |
| 293 | 贵州余农2号 |
| 294 | 梅柳10号 |
| 295 | 珍迁糯 |
| 296 | 红梅早 |
| 297 | 叶青伦 |
| 298 | 青桂矮5号 |
| 299 | 三黄占2号 |
| 300 | 双矮11 |
| 301 | 红辐(早）2 |
| 302 | 香丝苗2号 |
| 303 | 南丛3 |
| 304 | 桂野占2号 |
| 305 | 七青占 |
| 306 | 新青92 |
| 307 | 广科36 |
| 308 | 科青糯 |
| 309 | 汕优836-1 |
| 310 | 青珍1号 |
| 311 | 晚丰早1 |
| 312 | 南双矮 |
| 313 | 特三五1 |
| 314 | 玉粳占2 |
| 315 | 芦香占3 |
| 316 | 华丝占 |
| 317 | 莉粳红米 |
| 318 | 双粳占 |
| 319 | 特籼占13 |
| 320 | 锦山占 |
| 321 | 矮珍占 |
| 322 | 丰八占 |
| 323 | 矮秀占 |
| 324 | 银花占 |
| 325 | 中二软占 |
| 326 | 籼小占 |
| 327 | 粤香占 |
| 328 | 绿黄占 |
| 329 | 巴三占 |
| 330 | 二齐占 |
| 331 | 汉二糯 |
| 332 | 闷加黑丝 |
| 333 | 穗粳占 |
| 334 | 广源1 |
| 335 | 绿粳占1 |
| 336 | 源珍397 |
| 337 | 七澳占1 |
| 338 | 青华矮6 |
| 339 | 矮黑糯 |
| 340 | 新包矮 |
| 341 | 惠优占 |
| 342 | 广恢312 |
| 343 | 新铁大 |
| 344 | 胜泰1号 |
| 345 | 门较老4 |
| 346 | 七占 |
| 347 | 高穗种 |
| 348 | 糯仔 |
| 349 | 万宁谷(2) |
| 350 | 九占 |
| 351 | 黑蒂糯 |
| 352 | 日本种 |
| 353 | 新州占 |
| 354 | 铁秧快 |
| 355 | 番占种 |
| 356 | 香米 |
| 357 | 门教颖 |
| 358 | 长身红 |
| 359 | 白花 |
| 360 | 水粪旦 |
| 361 | 广西细米 |
| 362 | 大粒早熟 |
| 363 | 大白早 |
| 364 | 勿仔早 |
| 365 | 咸水占 |
| 366 | 襟得早 |
| 367 | 塔颈糯 |
| 368 | 旱稻 |
| 369 | 勾仔占 |
| 370 | 早禾白壳 |
| 371 | 大剑麻 |
| 372 | 早白仔 |
| 373 | 黑糯 |
| 374 | 麻壳占 |
| 375 | 龙川海禾 |
| 376 | 叶下长 |
| 377 | 龙牙仔 |
| 378 | 川早 |
| 379 | 1坑早 |
| 380 | JAMIR::IRGC 117765 |
| 381 | KALIBORO::IRGC 121006 |
| 382 | UCP 122::IRGC 8794 |
| 383 | UCP 122::IRGC 127871 |
| 384 | KALIBORO 2-2::IRGC 29353 |
| 385 | KALIBORO 26::IRGC 29355 |
| 386 | KALIBORO 41::IRGC 29356 |
| 387 | KALIBORO 80-3::IRGC 29358 |
| 388 | KALIBORO 138-2::IRGC 29361 |
| 389 | KALIBORO 704::IRGC 29368 |
| 390 | CIGEULIS |
| 391 | CIHERANG-SUBI |
| 392 | IR26 |
| 393 | IR36 |
| 394 | IR70 |
| 395 | IRRI123 |
| 396 | IRRI141 |
| 397 | IRRI154 |
| 398 | OM5629 |
| 399 | Saltolsinthwelatt |
| 400 | PSB-RC6 |
| 401 | PSB-RC8 |
| 402 | PSB-RC32 |
