# Supplementary material for: Genomic prediction of rice mesocotyl length indicative of directing seeding suitability using a half-sib hybrid population
Source: PLoS One. 2023 Apr 5;18(4):e0283989. doi: 10.1371/journal.pone.0283989 (PMC10075464; doi:10.1371/journal.pone.0283989)
Supplement: S3 Table — (DOCX) [file pone.0283989.s005.docx]

**Supplementary Table S3.** The best unbiased estimated values (BLUE) of mesocotyl length of 402 rice accessions and 401 hybrids.

|  | **ID** | **Designation** | **BLUE** |
| --- | --- | --- | --- |
| 1 |  | taifengA | 0.37 |
| 2 | YS003 | BHORO NATA::IRGC 31728 / IR 36 | 0.11 |
| 3 | YS004 | BHUA BALAM::IRGC 31729 / IR 36 | 0.76 |
| 4 | YS006 | CHAP SAIL::IRGC 31755 / IR 36 | 0.70 |
| 5 | YS007 | CHING JHORA::IRGC 31761 / IR 36 | 3.43 |
| 6 | YS008 | BAZAIL 1187::IRGC 32819 / IR 36 | 4.86 |
| 7 | YS009 | CHOTTO MOLLIK::IRGC 34687 / IR 36 | 4.08 |
| 8 | YS011 | ASHPALI::IRGC 36980 / IR 36 | 1.42 |
| 9 | YS012 | BHASAMANIK::IRGC 37014 / IR 36 | 0.69 |
| 10 | YS013 | BOALERDAT::IRGC 37020 / IR 36 | 0.16 |
| 11 | YS016 | BLACK 28-577::IRGC 37828 / IR 36 | 3.46 |
| 12 | YS017 | BLACK 28-578::IRGC 37829 / IR 36 | 2.60 |
| 13 | YS018 | BLACK 28-579::IRGC 37830 / IR 36 | 1.76 |
| 14 | YS019 | BR 52-87-1::IRGC 39191 / IR 36 | 3.66 |
| 15 | YS022 | CHOTO BAWALIA::IRGC 49268 / IR 36 | 4.95 |
| 16 | YS023 | BADI::IRGC 49300 / IR 36 | 4.68 |
| 17 | YS024 | BALA DHAL KUSRI::IRGC 49301 / IR 36 | 3.86 |
| 18 | YS025 | BARA LONA KURCHI::IRGC 49302 / IR 36 | 3.35 |
| 19 | YS027 | BATHAG::IRGC 49304 / IR 36 | 3.90 |
| 20 | YS028 | BHAHURI (AWN)::IRGC 49308 / IR 36 | 4.72 |
| 21 | YS029 | CASH::IRGC 49310 / IR 36 | 5.33 |
| 22 | YS030 | CHANGAI::IRGC 49311 / IR 36 | 5.87 |
| 23 | YS031 | BOROCHINAL::IRGC 133882 / IR 36 | 4.23 |
| 24 | YS032 | BHADOIA 689::IRGC 6536 / IR 36 | 4.67 |
| 25 | YS033 | APCHAYA::IRGC 26535 / IR 36 | 4.47 |
| 26 | YS034 | CHIKON::IRGC 31757 / IR 36 | 5.03 |
| 27 | YS036 | CHINRAVUCHI::IRGC 37048 / IR 36 | 3.46 |
| 28 | YS038 | AWNED 25-546::IRGC 37782 / IR 36 | 4.42 |
| 29 | YS039 | AWNED 25-550::IRGC 37786 / IR 36 | 3.95 |
| 30 | YS040 | BLACK 28-557::IRGC 37808 / IR 36 | 4.45 |
| 31 | YS041 | BLACK 28-571::IRGC 37822 / IR 36 | 4.03 |
| 32 | YS042 | BLACK 28-573::IRGC 37824 / IR 36 | 5.26 |
| 33 | YS043 | BHABANI (JABAIN)::IRGC 49266 / IR 36 | 5.74 |
| 34 | YS045 | CHAULA MAGHI::IRGC 49312 / IR 36 | 5.23 |
| 35 | YS046 | BR 10::IRGC 53457 / IR 36 | 5.39 |
| 36 | YS047 | BR 11::IRGC 53458 / IR 36 | 0.63 |
| 37 | YS048 | BARAROPA::IRGC 53470 / IR 36 | 2.56 |
| 38 | YS049 | AMAIRRA::IRGC 79305 / IR 36 | 4.48 |
| 39 | YS050 | BOROHAJI::IRGC 114597 / IR 36 | 0.01 |
| 40 | YS051 | BARAN BORO::IRGC 117434 / IR 36 | 4.02 |
| 41 | YS052 | CHANDARHAT::IRGC 121605 / IR 36 | 1.83 |
| 42 | YS053 | BAILA BORKI::IRGC 133693 / IR 36 | 3.11 |
| 43 | YS054 | BASFUL 714::IRGC 133714 / IR 36 | 4.65 |
| 44 | YS055 | MICOCHU::IRGC 121055 / IR 36 | 2.25 |
| 45 | YS057 | CHAPLAISH::IRGC 25846 / IR 36 | 5.04 |
| 46 | YS058 | DV 123::IRGC 117724 / IR 36 | 4.88 |
| 47 | YS059 | DJ 123::IRGC 117711 / IR 36 | 1.99 |
| 48 | YS060 | HOLOI BASH (SOLOI BASH)::IRGC 120969 / IR 36 | 0.85 |
| 49 | YS061 | TCHAMPA::IRGC 117585 / IR 36 | 4.14 |
| 50 | YS062 | JAMBALI BUSSA::IRGC 120995 / IR 36 | 5.73 |
| 51 | YS063 | KANGRI::IRGC 127488 / IR 36 | 5.53 |
| 52 | YS064 | RANRUWAN::IRGC 127763 / IR 36 | 4.73 |
| 53 | YS065 | KALAR KAR::IRGC 49737 / IR 36 | 0.53 |
| 54 | YS066 | TAK:IRGC 121582 / IR 36 | 5.69 |
| 55 | YS068 | KARENDOL::IRGC 37485 / IR 36 | 3.47 |
| 56 | YS070 | BOILAN:::IRGC 120899 / IR 36 | 3.96 |
| 57 | YS071 | DV 60::IRGC 8832 / IR 36 | 3.38 |
| 58 | YS073 | NATEL BORO::IRGC 127652 / IR 36 | 5.34 |
| 59 | YS074 | DJ 69::IRGC 127324 / IR 36 | 2.83 |
| 60 | YS075 | INDIA DULAR (NO ORDEM 7)::IRGC 127430 / IR 36 | 4.94 |
| 61 | YS076 | KOALARETA::IRGC 121624 / IR 36 | 5.04 |
| 62 | YS077 | ARC 11959::IRGC 121188 / IR 36 | 3.07 |
| 63 | YS078 | M 136-20::IRGC 35053 / IR 36 | 5.60 |
| 64 | YS079 | DA 27-C::IRGC 45628 / IR 36 | 4.40 |
| 65 | YS080 | T 65::IRGC 52773 / IR 36 | 4.84 |
| 66 | YS081 | NAYIMA::IRGC 121447 / IR 36 | 3.81 |
| 67 | YS082 | JC 148::IRGC 9069 / IR 36 | 5.49 |
| 68 | YS083 | ARC 7336::IRGC 127169 / IR 36 | 4.90 |
| 69 | YS084 | UPRH 58::IRGC 127879 / IR 36 | 5.10 |
| 70 | YS086 | GHAIYA::IRGC 128297 / IR 36 | 5.01 |
| 71 | YS088 | CHAMKA::IRGC 25844 / IR 36 | 4.62 |
| 72 | YS091 | BENA JHUPI::IRGC 127993 / IR 36 | 5.37 |
| 73 | YS092 | BLACK 28-576::IRGC 37827 / IR 36 | -0.07 |
| 74 | YS093 | BINNI DHAN::IRGC 26569 / IR 36 | 0.06 |
| 75 | YS097 | CHENGA SAIL::IRGC 31756 / IR 36 | 0.00 |
| 76 | YS098 | BRJ 1-13 B-55::IRGC 32582 / IR 36 | 2.44 |
| 77 | YS099 | AWNED 25-545::IRGC 37781 / IR 36 | 5.15 |
| 78 | YS100 | BLACK 28-553::IRGC 37804 / IR 36 | 1.07 |
| 79 | YS101 | BOW PAGAL::IRGC 43796 / IR 36 | 3.18 |
| 80 | YS102 | BR 194-1-2-1-2::IRGC 44013 / IR 36 | 1.17 |
| 81 | YS103 | BATA::IRGC 53473 / IR 36 | 3.65 |
| 82 | YS105 | AGUNI KARTIKSAIL::IRGC 77213 / IR 36 | 0.78 |
| 83 | YS107 | TA LAY | 0.32 |
| 84 | YS108 | HASAWI | 4.10 |
| 85 | YS110 | POKKALI 4 | 3.17 |
| 86 | YS112 | CHERIVIRUPPU | 0.05 |
| 87 | YS113 | GETU | 1.37 |
| 88 | YS114 | AKUNDI | 3.78 |
| 89 | YS115 | TAL MUGUR | 0.61 |
| 90 | YS116 | HASSAN TAREME | 2.55 |
| 91 | YS117 | BPI RI 2 | 3.70 |
| 92 | YS118 | ARC 18567 | 1.71 |
| 93 | YS122 | ORUMUNDAKAN | 1.46 |
| 94 | YS123 | WALIMBO | 1.57 |
| 95 | YS125 | RD 23 | 3.71 |
| 96 | YS126 | MSALIM JARO | 5.77 |
| 97 | YS128 | LAN SHENG | 0.97 |
| 98 | YS129 | IR 8866-30-3-1-4-2 | 1.03 |
| 99 | YS130 | CSR 11 | 0.78 |
| 100 | YS132 | PSBRC 50 | 2.29 |
| 101 | YS133 | POKKALI | -0.07 |
| 102 | YS134 | NSIC RC 106 | 1.63 |
| 103 | YS135 | IR 66946-3R-178-1-1 | 0.21 |
| 104 | YS137 | FL 478 | 0.20 |
| 105 | YS138 | IR 45427-2B-2-2B-1-1::G1 | -0.02 |
| 106 | YS139 | CSR 28 | 0.07 |
| 107 | YS140 | TCP-266-2-49-B-B-3 | 0.45 |
| 108 | YS141 | BRRI DHAN 53 | 3.41 |
| 109 | YS143 | POKKALI (8558) | 0.00 |
| 110 | YS144 | AGIR JAIL BIROI::IRGC 66763-1 | 0.11 |
| 111 | YS146 | CHAMKA | 4.20 |
| 112 | YS147 | AUS 362 | 1.59 |
| 113 | YS148 | IARI 5824::IRGC 14420 | 0.56 |
| 114 | YS149 | KH 998::IRGC 16948 | 0.77 |
| 115 | YS151 | DJ 99::IRGC 8465 | 2.28 |
| 116 | YS152 | DJ 68::IRGC 8833 | 4.28 |
| 117 | YS153 | DHULE BIZ::IRGC 31769 | 1.89 |
| 118 | YS154 | DULPI::IRGC 31779 | 2.27 |
| 119 | YS155 | DAINOS::IRGC 37053 | 0.48 |
| 120 | YS156 | DEPA::IRGC 37056 | 4.14 |
| 121 | YS158 | DUKSAIL::IRGC 37069 | 0.56 |
| 122 | YS159 | DULOBECH::IRGC 37071 | 0.23 |
| 123 | YS160 | ADT 8::IRGC 5921 | 3.11 |
| 124 | YS161 | JC 70::IRGC 9114 | 3.96 |
| 125 | YS163 | PATTI DHAN::IRGC 10019 | 3.52 |
| 126 | YS164 | TENGREY::IRGC 10186 | 3.55 |
| 127 | YS165 | KHURKIJOHA::IRGC 10187 | 4.12 |
| 128 | YS166 | TENGARI LOCAL::IRGC 10188 | 4.47 |
| 129 | YS167 | KRISHNA (CR 1-6)::IRGC 12889 | 0.46 |
| 130 | YS168 | RATNA (CR 44-11)::IRGC 12890 | 1.09 |
| 131 | YS169 | ASWATHI::IRGC 14783 | 0.99 |
| 132 | YS170 | CR 57-29::IRGC 15775 | 2.35 |
| 133 | YS171 | CR 60-10::IRGC 15777 | 0.48 |
| 134 | YS172 | KH 864::IRGC 16949 | 2.00 |
| 135 | YS173 | DA 5::IRGC 5855 | 4.27 |
| 136 | YS175 | GANGA SAGAR::IRGC 31650 | 3.15 |
| 137 | YS177 | CYLINDRICAL 30-662::IRGC 37900 | 3.52 |
| 138 | YS178 | DA 31-1-1::IRGC 37901 | 2.17 |
| 139 | YS180 | GANDI::IRGC 49186 | 4.82 |
| 140 | YS181 | BHASMANIK::IRGC 624 | 4.32 |
| 141 | YS183 | SR 26::IRGC 4918 | 4.25 |
| 142 | YS184 | ASGO::IRGC 6657 | 1.99 |
| 143 | YS185 | MAINAGURI::IRGC 10182 | 3.45 |
| 144 | YS186 | BONGABAR::IRGC 10184 | 5.29 |
| 145 | YS187 | IARI 10560::IRGC 14426 | 1.94 |
| 146 | YS189 | KI 63-4::IRGC 34767 | 0.54 |
| 147 | YS190 | KENDEL::IRGC 34991 | 1.02 |
| 148 | YS191 | SXC 199::IRGC 35173 | 4.03 |
| 149 | YS192 | TR 17::IRGC 36743 | 1.37 |
| 150 | YS193 | TELLA HAMSA::IRGC 39541 | 0.09 |
| 151 | YS194 | CRM 8-5708-3::IRGC 39583 | 0.52 |
| 152 | YS195 | OR 87-9::IRGC 39672 | 1.16 |
| 153 | YS196 | OR 117-22::IRGC 39682 | 3.01 |
| 154 | YS198 | CNM 17::IRGC 45378 | 1.38 |
| 155 | YS199 | GANGAJALIGHAT::IRGC 45674 | 3.14 |
| 156 | YS200 | JS 52-67::IRGC 45945 | 0.31 |
| 157 | YS201 | KAJALBHOG::IRGC 45951 | 3.79 |
| 158 | YS202 | KANTHAMALA::IRGC 46044 | 4.01 |
| 159 | YS203 | CO 11::IRGC 26841 | 0.14 |
| 160 | YS204 | MTU 8002::IRGC 28516 | 2.21 |
| 161 | YS205 | KI 68::IRGC 34768 | 0.34 |
| 162 | YS206 | BHADRA::IRGC 34858 | 4.16 |
| 163 | YS207 | TR 21::IRGC 36747 | 1.37 |
| 164 | YS208 | TR 25::IRGC 36750 | 2.93 |
| 165 | YS209 | CRHP 8::IRGC 36839 | 1.27 |
| 166 | YS210 | BM 45::IRGC 36840 | 3.32 |
| 167 | YS211 | AICRIP 111-17 (HPU 2181)::IRGC 39154 | 0.21 |
| 168 | YS212 | CN 539::IRGC 39234 | 4.71 |
| 169 | YS213 | IET 2300::IRGC 39268 | 1.98 |
| 170 | YS214 | IET 3262::IRGC 39275 | 2.58 |
| 171 | YS215 | KH 17854::IRGC 39476 | 1.15 |
| 172 | YS216 | P 4-1-11-21::IRGC 39524 | 1.28 |
| 173 | YS217 | TNAU 13471::IRGC 39542 | 0.50 |
| 174 | YS218 | BPT 1235::IRGC 39575 | 0.88 |
| 175 | YS219 | CNM 25::IRGC 39579 | 1.15 |
| 176 | YS220 | CN 44-33-3::IRGC 39581 | 1.62 |
| 177 | YS221 | CRK 30-40::IRGC 39582 | 2.59 |
| 178 | YS222 | CRM 8-5710-8::IRGC 39585 | 3.46 |
| 179 | YS223 | CRM 8-5712::IRGC 39586 | 4.83 |
| 180 | YS226 | CR 129-65::IRGC 39596 | 1.77 |
| 181 | YS227 | CR 189-62-12::IRGC 39618 | 1.23 |
| 182 | YS228 | HG 60-49::IRGC 39636 | 0.43 |
| 183 | YS229 | KRC 4::IRGC 39651 | 1.11 |
| 184 | YS230 | OR 83-26::IRGC 39671 | 0.39 |
| 185 | YS231 | OR 117-31::IRGC 39684 | 4.43 |
| 186 | YS232 | PAU 21-88-5::IRGC 39691 | 3.33 |
| 187 | YS233 | PAU 41-281-1-1::IRGC 39696 | 2.97 |
| 188 | YS234 | PAU 41-306-2-1::IRGC 39697 | 2.64 |
| 189 | YS235 | PAU 125-149-2::IRGC 39698 | 3.24 |
| 190 | YS236 | PUSA 33-30-18-3::IRGC 39707 | 1.66 |
| 191 | YS238 | RNR 56165-1::IRGC 39716 | 2.46 |
| 192 | YS239 | RP 825-28-7-1::IRGC 39765 | 0.88 |
| 193 | YS240 | RP 894-15-2-1-1::IRGC 39770 | 2.56 |
| 194 | YS241 | RP 894-61-1-3-7-2::IRGC 39771 | 3.58 |
| 195 | YS242 | RP 919-8-9-2-6-3::IRGC 39775 | 4.41 |
| 196 | YS243 | RP 932-4-11-3-4::IRGC 39777 | 2.49 |
| 197 | YS244 | RP 967-4-7-2-1::IRGC 39783 | 1.73 |
| 198 | YS245 | RP 967-4-7-2-7::IRGC 39785 | 1.44 |
| 199 | YS246 | RP 967-11-1-3-6::IRGC 39791 | 0.02 |
| 200 | YS248 | RP 974-133-7-29-16-9-4::IRGC 39814 | 2.65 |
| 201 | YS250 | RP 975-32-1-1-2::IRGC 39823 | -0.07 |
| 202 | YS251 | SS 55-304::IRGC 39854 | 0.19 |
| 203 | YS252 | TNAU 7583::IRGC 39860 | 1.07 |
| 204 | YS253 | TNAU 9485-7::IRGC 39864 | 3.37 |
| 205 | YS254 | OR 8023::IRGC 40016 | 0.32 |
| 206 | YS255 | UPR 96-1::IRGC 40065 | 4.21 |
| 207 | YS256 | RP 1303-80-1::IRGC 40141 | 2.19 |
| 208 | YS257 | BLACK GORA S N 32::IRGC 44819 | 5.50 |
| 209 | YS259 | BROWN GORA S N 12::IRGC 44832 | 4.36 |
| 210 | YS260 | BROWN GORA S N 68::IRGC 44852 | 5.25 |
| 211 | YS263 | AKANDARANGI::IRGC 44920 | 4.35 |
| 212 | YS264 | BARAHARIN::IRGC 45083 | 3.24 |
| 213 | YS265 | CHACHI::IRGC 45256 | 4.91 |
| 214 | YS266 | CR 115-76::IRGC 45413 | 0.46 |
| 215 | YS267 | DHIPISAIL::IRGC 45541 | 4.07 |
| 216 | YS268 | GODABELKI::IRGC 45696 | 1.90 |
| 217 | YS269 | HALDIJAB::IRGC 45746 | 3.99 |
| 218 | YS270 | HALDIJAM::IRGC 45747 | 4.57 |
| 219 | YS271 | HEERAMOTI::IRGC 45790 | 4.24 |
| 220 | YS272 | HELFULPI::IRGC 45792 | 3.60 |
| 221 | YS273 | IET 2233::IRGC 45839 | 0.43 |
| 222 | YS275 | KABRANONA::IRGC 45947 | 4.91 |
| 223 | YS279 | KALA RATA 1-24::IRGC 26913 | 1.08 |
| 224 | YS280 | CUL 688::IRGC 28559 | 2.28 |
| 225 | YS281 | TNAU 633::IRGC 28572 | 0.90 |
| 226 | YS282 | BR 7::IRGC 33995 | 5.70 |
| 227 | YS283 | DHUP SAIL::IRGC 34902 | 4.02 |
| 228 | YS284 | NARARI SABRI::IRGC 35060 | 3.77 |
| 229 | YS285 | K 140-52-3::IRGC 39511 | 2.30 |
| 230 | YS286 | P 33-C-30::IRGC 39526 | 3.04 |
| 231 | YS288 | CR 189-62-15::IRGC 39620 | -0.10 |
| 232 | YS289 | CUL 3/RATNA 55::IRGC 39627 | 0.32 |
| 233 | YS292 | PAU 41-10-1-3::IRGC 39694 | 3.36 |
| 234 | YS295 | RP 189-3::IRGC 39740 | 2.64 |
| 235 | YS298 | RP 825-92-4-16::IRGC 39767 | 2.25 |
| 236 | YS299 | RP 967-65-4-3-7::IRGC 39802 | 3.34 |
| 237 | YS302 | TNAU 13613::IRGC 39865 | 0.31 |
| 238 | YS303 | VADAGAON 416-34-2-6::IRGC 39870 | 1.68 |
| 239 | YS304 | BLACK GORA S N 19::IRGC 44818 | 5.00 |
| 240 | YS309 | CR 148-2623-215::IRGC 45437 | 0.91 |
| 241 | YS315 | CAROLINA::IRGC 3401 | 4.47 |
| 242 | YS317 | MUSHKAN 41::IRGC 6418 | 4.66 |
| 243 | YS318 | BELLO::IRGC 6658 | 4.87 |
| 244 | YS319 | DZ 60::IRGC 8558 | 4.41 |
| 245 | YS324 | SADRI::IRGC 33946 | 4.31 |
| 246 | YS325 | GUNDIL::IRGC 34544 | 0.89 |
| 247 | YS327 | KORGUT::IRGC 35003 | 2.20 |
| 248 | YS331 | MAROANTRANO::IRGC 97278 | 4.22 |
| 249 | YS332 | CYPRESS::IRGC 124359 | 3.70 |
| 250 | YS334 | MOROBEREKAN::IRGC 117272 | 2.10 |
| 251 | YS340 | BONDYL::IRGC 25842 | 0.19 |
| 252 | YS341 | CHAKKAL::IRGC 25843 | 1.03 |
| 253 | YS343 | AMBORO 1::IRGC 36970 | 1.17 |
| 254 | YS345 | BASIRAJ::IRGC 37003 | 1.39 |
| 255 | YS349 | BOTA::IRGC 37394 | 4.23 |
| 256 | YS350 | CHOTA BAZAL::IRGC 37407 | 4.05 |
| 257 | YS351 | ASAIL::IRGC 49052 | 0.26 |
| 258 | YS353 | BHOLANATH::IRGC 64767 | 4.71 |
| 259 | YS355 | BALAM 1::IRGC 77217 | 2.57 |
| 260 | YS356 | BOLONGA::IRGC 77224 | 1.02 |
| 261 | YS358 | BINI (BLACK)::IRGC 82142 | 3.52 |
| 262 | YS359 | BOILAM::IRGC 87170 | 4.62 |
| 263 | YS360 | CHINA IRRI::IRGC 87172 | 0.04 |
| 264 | YS363 | BENTOBALA::IRGC 26948 | 3.82 |
| 265 | YS364 | BAGIAMON 349::IRGC 6494 | 3.87 |
| 266 | YS365 | BAKOI::IRGC 25836 | 3.36 |
| 267 | YS368 | BASHPOR::IRGC 37002 | 4.68 |
| 268 | YS372 | BENA GACHYA::IRGC 37380 | 4.16 |
| 269 | YS374 | BUTUBALAM::IRGC 49165 | 1.98 |
| 270 | YS375 | CHAPLAIS::IRGC 49168 | 3.17 |
| 271 | YS376 | BADAI::IRGC 53464 | 3.97 |
| 272 | YS377 | BODESHI::IRGC 53481 | 5.04 |
| 273 | YS378 | BOITI::IRGC 53482 | 3.70 |
| 274 | YS379 | ASHA::IRGC 66765 | 3.29 |
| 275 | YS380 | BAMURA::IRGC 66770 | 2.62 |
| 276 | YS381 | BIR MAZLA::IRGC 66771 | 4.20 |
| 277 | YS382 | BHORIA AUS::IRGC 66830 | 4.04 |
| 278 | YS384 | BALAM 2::IRGC 77218 | 5.32 |
| 279 | YS385 | BHABANI::IRGC 77222 | 4.02 |
| 280 | YS386 | BAIGUN GATI::IRGC 79308 | 3.20 |
| 281 | YS387 | BARI BHADAR::IRGC 79309 | 4.22 |
| 282 | YS388 | BOUMAL::IRGC 79320 | 4.35 |
| 283 | YS390 | BINI (WHITE)::IRGC 82829 | 5.14 |
| 284 | YS392 | AGUNBAN::IRGC 25828 | 4.54 |
| 285 | YS394 | ARAI::IRGC 25830 | 3.23 |
| 286 | YS399 | BHOULAM::IRGC 37244 | 3.68 |
| 287 | YS400 | BADALI::IRGC 37362 | 4.38 |
| 288 | YS401 | BUSHRI::IRGC 37396 | 4.03 |
| 289 | YS402 | CHALAKI::IRGC 37398 | 4.55 |
| 290 | YS405 | ASSHINI::IRGC 53461 | 3.23 |
| 291 | YS607 | 三七早 | 0.38 |
| 292 | YS608 | 广场矮6号 | 1.13 |
| 293 | YS610 | 贵州余农2号 | -0.09 |
| 294 | YS611 | 梅柳10号 | 1.63 |
| 295 | YS612 | 珍迁糯 | 0.06 |
| 296 | YS613 | 红梅早 | 0.69 |
| 297 | YS614 | 叶青伦 | 0.23 |
| 298 | YS615 | 青桂矮5号 | 0.13 |
| 299 | YS616 | 三黄占2号 | 0.17 |
| 300 | YS617 | 双矮11 | 1.10 |
| 301 | YS618 | 红辐(早）2 | 0.73 |
| 302 | YS619 | 香丝苗2号 | 4.65 |
| 303 | YS620 | 南丛3 | -0.02 |
| 304 | YS621 | 桂野占2号 | 0.10 |
| 305 | YS622 | 七青占 | 3.72 |
| 306 | YS623 | 新青92 | 0.13 |
| 307 | YS624 | 广科36 | 0.27 |
| 308 | YS625 | 科青糯 | 1.63 |
| 309 | YS626 | 汕优836-1 | 2.43 |
| 310 | YS627 | 青珍1号 | 0.36 |
| 311 | YS628 | 晚丰早1 | 0.13 |
| 312 | YS629 | 南双矮 | -0.09 |
| 313 | YS630 | 特三五1 | 0.04 |
| 314 | YS631 | 玉粳占2 | 0.43 |
| 315 | YS632 | 芦香占3 | 0.42 |
| 316 | YS633 | 华丝占 | -0.04 |
| 317 | YS634 | 莉粳红米 | 0.00 |
| 318 | YS635 | 双粳占 | 0.19 |
| 319 | YS636 | 特籼占13 | 0.02 |
| 320 | YS637 | 锦山占 | 0.13 |
| 321 | YS638 | 矮珍占 | 0.17 |
| 322 | YS639 | 丰八占 | -0.04 |
| 323 | YS640 | 矮秀占 | 0.32 |
| 324 | YS641 | 银花占 | -0.10 |
| 325 | YS642 | 中二软占 | 2.28 |
| 326 | YS643 | 籼小占 | 1.39 |
| 327 | YS645 | 粤香占 | 2.15 |
| 328 | YS646 | 绿黄占 | 0.10 |
| 329 | YS647 | 巴三占 | 3.92 |
| 330 | YS648 | 二齐占 | -0.09 |
| 331 | YS649 | 汉二糯 | 0.01 |
| 332 | YS650 | 闷加黑丝 | -0.08 |
| 333 | YS651 | 穗粳占 | 1.81 |
| 334 | YS652 | 广源1 | 1.08 |
| 335 | YS653 | 绿粳占1 | 0.27 |
| 336 | YS654 | 源珍397 | 0.61 |
| 337 | YS655 | 七澳占1 | 0.34 |
| 338 | YS656 | 青华矮6 | 0.61 |
| 339 | YS658 | 矮黑糯 | 0.23 |
| 340 | YS659 | 新包矮 | 2.50 |
| 341 | YS660 | 惠优占 | 0.75 |
| 342 | YS661 | 广恢312 | 0.26 |
| 343 | YS662 | 新铁大 | -0.02 |
| 344 | YS663 | 胜泰1号 | 0.62 |
| 345 | YS664 | 门较老4 | 0.53 |
| 346 | YS665 | 七占 | 4.41 |
| 347 | YS667 | 高穗种 | 1.73 |
| 348 | YS669 | 糯仔 | 1.24 |
| 349 | YS670 | 万宁谷(2) | 3.76 |
| 350 | YS671 | 九占 | 2.83 |
| 351 | YS672 | 黑蒂糯 | 2.11 |
| 352 | YS673 | 日本种 | 1.03 |
| 353 | YS676 | 新州占 | 0.39 |
| 354 | YS677 | 铁秧快 | 3.45 |
| 355 | YS678 | 番占种 | 3.45 |
| 356 | YS679 | 香米 | 0.17 |
| 357 | YS680 | 门教颖 | 2.28 |
| 358 | YS681 | 长身红 | 2.31 |
| 359 | YS682 | 白花 | 0.00 |
| 360 | YS683 | 水粪旦 | 1.38 |
| 361 | YS684 | 广西细米 | 0.21 |
| 362 | YS685 | 大粒早熟 | 4.75 |
| 363 | YS686 | 大白早 | 0.37 |
| 364 | YS687 | 勿仔早 | -0.14 |
| 365 | YS688 | 咸水占 | 0.10 |
| 366 | YS690 | 襟得早 | 1.18 |
| 367 | YS691 | 塔颈糯 | 2.55 |
| 368 | YS692 | 旱稻 | 4.80 |
| 369 | YS693 | 勾仔占 | -0.10 |
| 370 | YS694 | 早禾白壳 | 0.58 |
| 371 | YS695 | 大剑麻 | 0.79 |
| 372 | YS696 | 早白仔 | 1.98 |
| 373 | YS697 | 黑糯 | 0.77 |
| 374 | YS698 | 麻壳占 | 0.12 |
| 375 | YS700 | 龙川海禾 | 0.00 |
| 376 | YS701 | 叶下长 | 0.87 |
| 377 | YS702 | 龙牙仔 | -0.06 |
| 378 | YS705 | 川早 | 0.00 |
| 379 | YS706 | 1坑早 | 0.34 |
| 380 | YS707 | JAMIR::IRGC 117765 | 4.36 |
| 381 | YS708 | KALIBORO::IRGC 121006 | 2.10 |
| 382 | YS709 | UCP 122::IRGC 8794 | 4.17 |
| 383 | YS710 | UCP 122::IRGC 127871 | 4.82 |
| 384 | YS711 | KALIBORO 2-2::IRGC 29353 | 2.46 |
| 385 | YS712 | KALIBORO 26::IRGC 29355 | 0.29 |
| 386 | YS713 | KALIBORO 41::IRGC 29356 | 0.31 |
| 387 | YS714 | KALIBORO 80-3::IRGC 29358 | 3.20 |
| 388 | YS715 | KALIBORO 138-2::IRGC 29361 | 2.19 |
| 389 | YS716 | KALIBORO 704::IRGC 29368 | 0.29 |
| 390 | YS719 | CIGEULIS | 0.24 |
| 391 | YS720 | CIHERANG-SUBI | 0.24 |
| 392 | YS726 | IR26 | 3.40 |
| 393 | YS728 | IR36 | 3.49 |
| 394 | YS737 | IR70 | 2.80 |
| 395 | YS754 | IRRI123 | 4.01 |
| 396 | YS758 | IRRI141 | 1.54 |
| 397 | YS765 | IRRI154 | 3.37 |
| 398 | YS783 | OM5629 | 3.50 |
| 399 | YS790 | Saltolsinthwelatt | 2.41 |
| 400 | YS803 | PSB-RC6 | 2.66 |
| 401 | YS805 | PSB-RC8 | 3.43 |
| 402 | YS820 | PSB-RC32 | 4.30 |

|  | **ID** | **BLUE** |
| --- | --- | --- |
| 1 | YS003_A | 0.36 |
| 2 | YS004_A | 2.09 |
| 3 | YS006_A | 3.46 |
| 4 | YS007_A | 3.41 |
| 5 | YS008_A | 4.37 |
| 6 | YS009_A | 4.40 |
| 7 | YS011_A | 0.46 |
| 8 | YS012_A | 0.85 |
| 9 | YS013_A | 1.73 |
| 10 | YS016_A | 4.84 |
| 11 | YS017_A | 0.46 |
| 12 | YS018_A | 0.34 |
| 13 | YS019_A | 0.64 |
| 14 | YS022_A | 4.14 |
| 15 | YS023_A | 4.74 |
| 16 | YS024_A | 4.53 |
| 17 | YS025_A | 3.82 |
| 18 | YS027_A | 3.29 |
| 19 | YS028_A | 4.05 |
| 20 | YS029_A | 3.79 |
| 21 | YS030_A | 5.01 |
| 22 | YS031_A | 2.00 |
| 23 | YS032_A | 1.85 |
| 24 | YS033_A | 4.14 |
| 25 | YS034_A | 4.25 |
| 26 | YS036_A | 4.66 |
| 27 | YS038_A | 3.79 |
| 28 | YS039_A | 3.94 |
| 29 | YS040_A | 3.70 |
| 30 | YS041_A | 3.47 |
| 31 | YS042_A | 5.12 |
| 32 | YS043_A | 4.92 |
| 33 | YS045_A | 4.79 |
| 34 | YS046_A | 4.96 |
| 35 | YS047_A | 0.58 |
| 36 | YS048_A | 1.74 |
| 37 | YS049_A | 4.07 |
| 38 | YS050_A | 1.90 |
| 39 | YS051_A | 1.67 |
| 40 | YS052_A | 2.49 |
| 41 | YS053_A | 3.99 |
| 42 | YS054_A | 4.94 |
| 43 | YS055_A | 3.89 |
| 44 | YS057_A | 1.82 |
| 45 | YS058_A | 4.10 |
| 46 | YS059_A | 2.82 |
| 47 | YS060_A | 2.01 |
| 48 | YS061_A | 2.93 |
| 49 | YS062_A | 2.46 |
| 50 | YS063_A | 2.70 |
| 51 | YS064_A | 1.04 |
| 52 | YS065_A | 0.56 |
| 53 | YS066_A | 4.93 |
| 54 | YS068_A | 0.85 |
| 55 | YS070_A | 3.12 |
| 56 | YS071_A | 3.36 |
| 57 | YS073_A | 4.98 |
| 58 | YS074_A | 3.36 |
| 59 | YS075_A | 3.88 |
| 60 | YS076_A | 4.56 |
| 61 | YS077_A | 4.59 |
| 62 | YS078_A | 4.66 |
| 63 | YS079_A | 4.60 |
| 64 | YS080_A | 2.34 |
| 65 | YS081_A | 1.09 |
| 66 | YS082_A | 1.92 |
| 67 | YS083_A | 4.87 |
| 68 | YS084_A | 5.10 |
| 69 | YS086_A | 2.02 |
| 70 | YS088_A | 5.04 |
| 71 | YS091_A | 4.55 |
| 72 | YS092_A | 1.40 |
| 73 | YS093_A | 1.37 |
| 74 | YS097_A | 0.01 |
| 75 | YS098_A | 0.41 |
| 76 | YS099_A | 0.92 |
| 77 | YS100_A | 2.34 |
| 78 | YS101_A | 0.44 |
| 79 | YS102_A | 2.83 |
| 80 | YS103_A | 3.96 |
| 81 | YS105_A | 0.24 |
| 82 | YS107_A | 2.38 |
| 83 | YS108_A | 3.35 |
| 84 | YS110_A | 4.22 |
| 85 | YS112_A | 2.64 |
| 86 | YS113_A | 3.95 |
| 87 | YS114_A | 1.70 |
| 88 | YS115_A | 2.20 |
| 89 | YS116_A | 3.84 |
| 90 | YS117_A | 0.38 |
| 91 | YS118_A | 0.06 |
| 92 | YS122_A | 2.77 |
| 93 | YS123_A | 3.26 |
| 94 | YS125_A | 1.24 |
| 95 | YS126_A | 5.61 |
| 96 | YS128_A | 1.62 |
| 97 | YS129_A | 2.05 |
| 98 | YS130_A | 0.38 |
| 99 | YS132_A | 0.83 |
| 100 | YS133_A | 2.12 |
| 101 | YS134_A | 0.62 |
| 102 | YS135_A | 0.15 |
| 103 | YS137_A | 0.13 |
| 104 | YS138_A | 0.18 |
| 105 | YS139_A | 0.05 |
| 106 | YS140_A | 2.55 |
| 107 | YS141_A | 1.06 |
| 108 | YS143_A | 0.11 |
| 109 | YS144_A | 2.07 |
| 110 | YS146_A | 3.48 |
| 111 | YS147_A | 2.93 |
| 112 | YS148_A | 0.29 |
| 113 | YS149_A | 1.77 |
| 114 | YS151_A | 0.47 |
| 115 | YS152_A | 4.26 |
| 116 | YS153_A | 3.26 |
| 117 | YS154_A | 4.23 |
| 118 | YS155_A | 2.50 |
| 119 | YS156_A | 3.97 |
| 120 | YS158_A | 3.97 |
| 121 | YS159_A | 3.15 |
| 122 | YS160_A | 3.80 |
| 123 | YS161_A | 4.36 |
| 124 | YS163_A | 4.08 |
| 125 | YS164_A | 4.78 |
| 126 | YS165_A | 4.82 |
| 127 | YS166_A | 4.40 |
| 128 | YS167_A | 3.65 |
| 129 | YS168_A | 0.52 |
| 130 | YS169_A | 0.30 |
| 131 | YS170_A | 0.92 |
| 132 | YS171_A | 0.57 |
| 133 | YS172_A | 2.68 |
| 134 | YS173_A | 4.26 |
| 135 | YS175_A | 3.72 |
| 136 | YS177_A | 4.72 |
| 137 | YS178_A | 3.52 |
| 138 | YS180_A | 4.82 |
| 139 | YS181_A | 4.24 |
| 140 | YS183_A | 4.70 |
| 141 | YS184_A | 1.83 |
| 142 | YS185_A | 4.65 |
| 143 | YS186_A | 5.16 |
| 144 | YS187_A | 0.09 |
| 145 | YS189_A | 0.98 |
| 146 | YS190_A | 1.28 |
| 147 | YS191_A | 1.71 |
| 148 | YS192_A | 0.21 |
| 149 | YS193_A | 3.65 |
| 150 | YS194_A | 0.36 |
| 151 | YS195_A | 2.52 |
| 152 | YS196_A | 4.33 |
| 153 | YS198_A | 1.33 |
| 154 | YS199_A | 3.90 |
| 155 | YS200_A | 0.32 |
| 156 | YS201_A | 4.64 |
| 157 | YS202_A | 4.40 |
| 158 | YS203_A | 0.06 |
| 159 | YS204_A | 2.73 |
| 160 | YS205_A | 0.45 |
| 161 | YS206_A | 2.08 |
| 162 | YS207_A | 0.35 |
| 163 | YS208_A | 0.61 |
| 164 | YS209_A | 0.41 |
| 165 | YS210_A | 0.50 |
| 166 | YS211_A | 0.08 |
| 167 | YS212_A | 4.28 |
| 168 | YS213_A | 0.72 |
| 169 | YS214_A | 0.45 |
| 170 | YS215_A | 2.16 |
| 171 | YS216_A | 0.17 |
| 172 | YS217_A | 0.47 |
| 173 | YS218_A | 0.23 |
| 174 | YS219_A | 0.26 |
| 175 | YS220_A | 2.34 |
| 176 | YS221_A | 0.27 |
| 177 | YS222_A | 3.74 |
| 178 | YS223_A | 4.54 |
| 179 | YS226_A | 0.19 |
| 180 | YS227_A | 0.53 |
| 181 | YS228_A | 0.20 |
| 182 | YS229_A | 0.21 |
| 183 | YS230_A | 2.66 |
| 184 | YS231_A | 4.32 |
| 185 | YS232_A | 0.33 |
| 186 | YS233_A | 0.27 |
| 187 | YS234_A | 0.66 |
| 188 | YS235_A | 0.49 |
| 189 | YS236_A | 0.13 |
| 190 | YS238_A | 0.34 |
| 191 | YS239_A | 4.69 |
| 192 | YS240_A | 0.34 |
| 193 | YS241_A | 0.36 |
| 194 | YS242_A | 2.01 |
| 195 | YS243_A | 0.49 |
| 196 | YS244_A | 0.16 |
| 197 | YS245_A | 3.09 |
| 198 | YS246_A | 2.79 |
| 199 | YS248_A | 0.59 |
| 200 | YS250_A | 0.10 |
| 201 | YS251_A | 0.12 |
| 202 | YS252_A | 0.49 |
| 203 | YS253_A | 4.25 |
| 204 | YS254_A | 2.75 |
| 205 | YS255_A | 1.03 |
| 206 | YS256_A | 2.42 |
| 207 | YS257_A | 1.76 |
| 208 | YS259_A | 5.07 |
| 209 | YS260_A | 2.78 |
| 210 | YS263_A | 4.70 |
| 211 | YS264_A | 3.76 |
| 212 | YS265_A | 5.37 |
| 213 | YS266_A | 2.21 |
| 214 | YS267_A | 4.61 |
| 215 | YS268_A | 3.65 |
| 216 | YS269_A | 4.50 |
| 217 | YS270_A | 4.78 |
| 218 | YS271_A | 0.47 |
| 219 | YS272_A | 5.18 |
| 220 | YS273_A | 0.39 |
| 221 | YS275_A | 5.16 |
| 222 | YS279_A | 0.56 |
| 223 | YS280_A | 4.26 |
| 224 | YS281_A | 0.10 |
| 225 | YS282_A | 5.41 |
| 226 | YS283_A | 2.71 |
| 227 | YS284_A | 1.50 |
| 228 | YS285_A | 3.69 |
| 229 | YS286_A | 0.53 |
| 230 | YS288_A | 0.25 |
| 231 | YS289_A | 1.81 |
| 232 | YS292_A | 0.39 |
| 233 | YS295_A | 0.40 |
| 234 | YS298_A | 0.44 |
| 235 | YS299_A | 0.72 |
| 236 | YS302_A | 0.02 |
| 237 | YS303_A | 0.63 |
| 238 | YS304_A | 0.78 |
| 239 | YS309_A | 0.08 |
| 240 | YS315_A | 1.37 |
| 241 | YS317_A | 4.62 |
| 242 | YS318_A | 4.91 |
| 243 | YS319_A | 3.95 |
| 244 | YS324_A | 4.63 |
| 245 | YS325_A | 0.98 |
| 246 | YS327_A | 3.72 |
| 247 | YS331_A | 4.37 |
| 248 | YS332_A | 4.50 |
| 249 | YS334_A | 0.97 |
| 250 | YS340_A | 3.11 |
| 251 | YS341_A | 3.61 |
| 252 | YS343_A | 1.08 |
| 253 | YS345_A | 3.51 |
| 254 | YS349_A | 3.62 |
| 255 | YS350_A | 4.50 |
| 256 | YS351_A | 2.00 |
| 257 | YS353_A | 4.45 |
| 258 | YS355_A | 3.47 |
| 259 | YS356_A | 1.24 |
| 260 | YS358_A | 5.12 |
| 261 | YS359_A | 4.69 |
| 262 | YS360_A | 1.76 |
| 263 | YS363_A | 4.27 |
| 264 | YS364_A | 4.99 |
| 265 | YS365_A | 4.37 |
| 266 | YS368_A | 4.38 |
| 267 | YS372_A | 4.71 |
| 268 | YS374_A | 1.93 |
| 269 | YS375_A | 3.02 |
| 270 | YS376_A | 3.73 |
| 271 | YS377_A | 0.49 |
| 272 | YS378_A | 1.36 |
| 273 | YS379_A | 1.23 |
| 274 | YS380_A | 3.13 |
| 275 | YS381_A | 4.37 |
| 276 | YS382_A | 1.71 |
| 277 | YS384_A | 5.28 |
| 278 | YS385_A | 3.84 |
| 279 | YS386_A | 0.62 |
| 280 | YS387_A | 1.98 |
| 281 | YS388_A | 1.32 |
| 282 | YS390_A | 1.66 |
| 283 | YS392_A | 1.34 |
| 284 | YS394_A | 2.62 |
| 285 | YS399_A | 0.75 |
| 286 | YS400_A | 4.32 |
| 287 | YS401_A | 4.31 |
| 288 | YS402_A | 4.78 |
| 289 | YS405_A | 1.70 |
| 290 | YS607_A | 2.33 |
| 291 | YS608_A | 3.05 |
| 292 | YS610_A | -0.06 |
| 293 | YS611_A | 0.46 |
| 294 | YS612_A | 0.22 |
| 295 | YS613_A | 2.48 |
| 296 | YS614_A | 0.19 |
| 297 | YS615_A | 0.20 |
| 298 | YS616_A | 0.26 |
| 299 | YS617_A | 2.67 |
| 300 | YS618_A | 2.50 |
| 301 | YS619_A | 4.01 |
| 302 | YS620_A | 0.64 |
| 303 | YS621_A | 0.18 |
| 304 | YS622_A | 1.41 |
| 305 | YS623_A | 0.07 |
| 306 | YS624_A | 0.32 |
| 307 | YS625_A | 0.28 |
| 308 | YS626_A | 0.01 |
| 309 | YS627_A | 0.19 |
| 310 | YS628_A | 0.25 |
| 311 | YS629_A | -0.04 |
| 312 | YS630_A | 1.28 |
| 313 | YS631_A | 0.23 |
| 314 | YS632_A | 0.23 |
| 315 | YS633_A | -0.10 |
| 316 | YS634_A | -0.05 |
| 317 | YS635_A | -0.06 |
| 318 | YS636_A | 0.06 |
| 319 | YS637_A | 0.03 |
| 320 | YS638_A | -0.02 |
| 321 | YS639_A | 0.15 |
| 322 | YS640_A | 0.21 |
| 323 | YS641_A | 0.48 |
| 324 | YS642_A | 0.15 |
| 325 | YS643_A | 0.29 |
| 326 | YS645_A | 2.98 |
| 327 | YS646_A | 0.12 |
| 328 | YS647_A | 0.79 |
| 329 | YS648_A | 0.13 |
| 330 | YS649_A | 2.74 |
| 331 | YS650_A | 0.12 |
| 332 | YS651_A | 0.49 |
| 333 | YS652_A | 0.47 |
| 334 | YS653_A | 0.25 |
| 335 | YS654_A | 0.25 |
| 336 | YS655_A | 0.60 |
| 337 | YS656_A | 0.24 |
| 338 | YS658_A | 0.39 |
| 339 | YS659_A | 0.32 |
| 340 | YS660_A | 0.22 |
| 341 | YS661_A | 0.10 |
| 342 | YS662_A | 0.32 |
| 343 | YS663_A | 0.35 |
| 344 | YS664_A | 3.29 |
| 345 | YS665_A | 4.34 |
| 346 | YS667_A | 2.68 |
| 347 | YS669_A | 3.26 |
| 348 | YS670_A | 4.29 |
| 349 | YS671_A | 4.25 |
| 350 | YS672_A | 3.03 |
| 351 | YS673_A | 0.36 |
| 352 | YS676_A | 2.78 |
| 353 | YS677_A | 0.88 |
| 354 | YS678_A | 3.66 |
| 355 | YS679_A | 2.68 |
| 356 | YS680_A | 0.92 |
| 357 | YS681_A | 3.92 |
| 358 | YS682_A | 2.74 |
| 359 | YS683_A | 4.41 |
| 360 | YS684_A | 3.36 |
| 361 | YS685_A | 5.37 |
| 362 | YS686_A | 3.85 |
| 363 | YS687_A | 0.17 |
| 364 | YS688_A | 0.24 |
| 365 | YS690_A | 3.77 |
| 366 | YS691_A | 3.83 |
| 367 | YS692_A | 0.96 |
| 368 | YS693_A | 1.62 |
| 369 | YS694_A | 0.29 |
| 370 | YS695_A | 1.11 |
| 371 | YS696_A | 4.14 |
| 372 | YS697_A | 3.37 |
| 373 | YS698_A | 0.41 |
| 374 | YS700_A | 1.80 |
| 375 | YS701_A | 3.43 |
| 376 | YS702_A | 0.04 |
| 377 | YS705_A | 3.20 |
| 378 | YS706_A | 0.40 |
| 379 | YS707_A | 4.35 |
| 380 | YS708_A | 4.12 |
| 381 | YS709_A | 3.89 |
| 382 | YS710_A | 4.81 |
| 383 | YS711_A | 0.62 |
| 384 | YS712_A | 2.99 |
| 385 | YS713_A | 0.77 |
| 386 | YS714_A | 0.98 |
| 387 | YS715_A | 0.80 |
| 388 | YS716_A | 1.07 |
| 389 | YS719_A | 0.44 |
| 390 | YS720_A | 0.42 |
| 391 | YS726_A | 1.91 |
| 392 | YS728_A | 0.87 |
| 393 | YS737_A | 0.69 |
| 394 | YS754_A | 0.51 |
| 395 | YS758_A | 0.64 |
| 396 | YS765_A | 0.57 |
| 397 | YS783_A | 1.75 |
| 398 | YS790_A | 3.02 |
| 399 | YS803_A | 0.76 |
| 400 | YS805_A | 3.23 |
| 401 | YS820_A | 4.41 |
