# Supplementary material for: Genomic prediction of rice mesocotyl length indicative of directing seeding suitability using a half-sib hybrid population
Source: PLoS One. 2023 Apr 5;18(4):e0283989. doi: 10.1371/journal.pone.0283989 (PMC10075464; doi:10.1371/journal.pone.0283989)
Supplement: S4 Table — Mid-parent value is the average best linear unbiased estimates (BLUE) of mesocotyl length of parents of the hybrids. d-value is the difference of BLUE between hybrid and its mid-parent value. a-value is absolute value of the difference of BLUE between parents. Hp is d-value / a-value. High parent heterosis (HPH) represents the Hp of hybrid was over 1. Mid-parent heterosis (MPH) represents the Hp of hybrid was over 0 and below 1 (including 1). Low-parent heterosis (LPH) represents the Hp of hybrid was over -1(including -1) and below 0. Hybrid inferiority (HI) represents the Hp of hybrid was below -1. (DOCX) [file pone.0283989.s006.docx]

**Supplementary Table S4.** Heterosis analysis of 401 hybrids.

|  | **F1-ID** | **BLUE** | **Mid-parent value** | **d-value** | **a-value** | **Hp(d/a)** | **Heterosis** |
| --- | --- | --- | --- | --- | --- | --- | --- |
| 1 | YS003_A | 0.36 | 0.24 | 0.12 | 0.13 | 0.91 | MPH |
| 2 | YS004_A | 2.09 | 0.57 | 1.52 | 0.19 | 7.86 | HPH |
| 3 | YS006_A | 3.46 | 0.54 | 2.92 | 0.17 | 17.67 | HPH |
| 4 | YS007_A | 3.41 | 1.90 | 1.51 | 1.53 | 0.99 | MPH |
| 5 | YS008_A | 4.37 | 2.61 | 1.76 | 2.24 | 0.78 | MPH |
| 6 | YS009_A | 4.40 | 2.23 | 2.18 | 1.85 | 1.18 | HPH |
| 7 | YS011_A | 0.46 | 0.90 | -0.44 | 0.52 | -0.84 | LPH |
| 8 | YS012_A | 0.85 | 0.53 | 0.32 | 0.16 | 2.02 | HPH |
| 9 | YS013_A | 1.73 | 0.27 | 1.47 | 0.11 | 13.74 | HPH |
| 10 | YS016_A | 4.84 | 1.92 | 2.92 | 1.54 | 1.89 | HPH |
| 11 | YS017_A | 0.46 | 1.49 | -1.02 | 1.11 | -0.92 | LPH |
| 12 | YS018_A | 0.34 | 1.07 | -0.73 | 0.70 | -1.05 | HI |
| 13 | YS019_A | 0.64 | 2.02 | -1.38 | 1.64 | -0.84 | LPH |
| 14 | YS022_A | 4.14 | 2.66 | 1.48 | 2.29 | 0.65 | MPH |
| 15 | YS023_A | 4.74 | 2.53 | 2.21 | 2.15 | 1.03 | HPH |
| 16 | YS024_A | 4.53 | 2.12 | 2.41 | 1.75 | 1.38 | HPH |
| 17 | YS025_A | 3.82 | 1.86 | 1.96 | 1.49 | 1.32 | HPH |
| 18 | YS027_A | 3.29 | 2.14 | 1.16 | 1.76 | 0.66 | MPH |
| 19 | YS028_A | 4.05 | 2.55 | 1.51 | 2.17 | 0.69 | MPH |
| 20 | YS029_A | 3.79 | 2.85 | 0.93 | 2.48 | 0.38 | MPH |
| 21 | YS030_A | 5.01 | 3.12 | 1.89 | 2.75 | 0.69 | MPH |
| 22 | YS031_A | 2.00 | 2.30 | -0.31 | 1.93 | -0.16 | LPH |
| 23 | YS032_A | 1.85 | 2.52 | -0.67 | 2.15 | -0.31 | LPH |
| 24 | YS033_A | 4.14 | 2.42 | 1.72 | 2.05 | 0.84 | MPH |
| 25 | YS034_A | 4.25 | 2.70 | 1.54 | 2.33 | 0.66 | MPH |
| 26 | YS036_A | 4.66 | 1.92 | 2.74 | 1.54 | 1.78 | HPH |
| 27 | YS038_A | 3.79 | 2.39 | 1.40 | 2.02 | 0.69 | MPH |
| 28 | YS039_A | 3.94 | 2.16 | 1.78 | 1.79 | 1.00 | MPH |
| 29 | YS040_A | 3.70 | 2.41 | 1.28 | 2.04 | 0.63 | MPH |
| 30 | YS041_A | 3.47 | 2.20 | 1.27 | 1.83 | 0.69 | MPH |
| 31 | YS042_A | 5.12 | 2.81 | 2.31 | 2.44 | 0.95 | MPH |
| 32 | YS043_A | 4.92 | 3.06 | 1.86 | 2.68 | 0.69 | MPH |
| 33 | YS045_A | 4.79 | 2.80 | 1.99 | 2.43 | 0.82 | MPH |
| 34 | YS046_A | 4.96 | 2.88 | 2.08 | 2.51 | 0.83 | MPH |
| 35 | YS047_A | 0.58 | 0.50 | 0.08 | 0.13 | 0.60 | MPH |
| 36 | YS048_A | 1.74 | 1.47 | 0.27 | 1.09 | 0.25 | MPH |
| 37 | YS049_A | 4.07 | 2.43 | 1.64 | 2.06 | 0.80 | MPH |
| 38 | YS050_A | 1.90 | 0.19 | 1.71 | 0.18 | 9.46 | HPH |
| 39 | YS051_A | 1.67 | 2.19 | -0.53 | 1.82 | -0.29 | LPH |
| 40 | YS052_A | 2.49 | 1.10 | 1.39 | 0.73 | 1.90 | HPH |
| 41 | YS053_A | 3.99 | 1.74 | 2.25 | 1.37 | 1.64 | HPH |
| 42 | YS054_A | 4.94 | 2.51 | 2.42 | 2.14 | 1.13 | HPH |
| 43 | YS055_A | 3.89 | 1.31 | 2.58 | 0.94 | 2.76 | HPH |
| 44 | YS057_A | 1.82 | 2.71 | -0.89 | 2.34 | -0.38 | LPH |
| 45 | YS058_A | 4.10 | 2.63 | 1.47 | 2.25 | 0.65 | MPH |
| 46 | YS059_A | 2.82 | 1.18 | 1.64 | 0.81 | 2.03 | HPH |
| 47 | YS060_A | 2.01 | 0.61 | 1.40 | 0.24 | 5.85 | HPH |
| 48 | YS061_A | 2.93 | 2.25 | 0.68 | 1.88 | 0.36 | MPH |
| 49 | YS062_A | 2.46 | 3.05 | -0.59 | 2.68 | -0.22 | LPH |
| 50 | YS063_A | 2.70 | 2.95 | -0.25 | 2.58 | -0.10 | LPH |
| 51 | YS064_A | 1.04 | 2.55 | -1.51 | 2.18 | -0.69 | LPH |
| 52 | YS065_A | 0.56 | 0.45 | 0.11 | 0.08 | 1.45 | HPH |
| 53 | YS066_A | 4.93 | 3.03 | 1.90 | 2.66 | 0.72 | MPH |
| 54 | YS068_A | 0.85 | 1.92 | -1.07 | 1.55 | -0.69 | LPH |
| 55 | YS070_A | 3.12 | 2.17 | 0.95 | 1.80 | 0.53 | MPH |
| 56 | YS071_A | 3.36 | 1.87 | 1.48 | 1.50 | 0.99 | MPH |
| 57 | YS073_A | 4.98 | 2.85 | 2.12 | 2.48 | 0.86 | MPH |
| 58 | YS074_A | 3.36 | 1.60 | 1.76 | 1.23 | 1.43 | HPH |
| 59 | YS075_A | 3.88 | 2.66 | 1.22 | 2.29 | 0.53 | MPH |
| 60 | YS076_A | 4.56 | 2.71 | 1.85 | 2.33 | 0.79 | MPH |
| 61 | YS077_A | 4.59 | 1.72 | 2.87 | 1.35 | 2.13 | HPH |
| 62 | YS078_A | 4.66 | 2.99 | 1.68 | 2.61 | 0.64 | MPH |
| 63 | YS079_A | 4.60 | 2.39 | 2.21 | 2.01 | 1.10 | HPH |
| 64 | YS080_A | 2.34 | 2.61 | -0.26 | 2.23 | -0.12 | LPH |
| 65 | YS081_A | 1.09 | 2.09 | -1.00 | 1.72 | -0.58 | LPH |
| 66 | YS082_A | 1.92 | 2.93 | -1.01 | 2.56 | -0.39 | LPH |
| 67 | YS083_A | 4.87 | 2.63 | 2.23 | 2.26 | 0.99 | MPH |
| 68 | YS084_A | 5.10 | 2.74 | 2.36 | 2.37 | 1.00 | MPH |
| 69 | YS086_A | 2.02 | 2.69 | -0.67 | 2.32 | -0.29 | LPH |
| 70 | YS088_A | 5.04 | 2.50 | 2.55 | 2.12 | 1.20 | HPH |
| 71 | YS091_A | 4.55 | 2.87 | 1.68 | 2.50 | 0.67 | MPH |
| 72 | YS092_A | 1.40 | 0.15 | 1.25 | 0.22 | 5.67 | HPH |
| 73 | YS093_A | 1.37 | 0.21 | 1.16 | 0.16 | 7.29 | HPH |
| 74 | YS097_A | 0.01 | 0.19 | -0.18 | 0.18 | -0.96 | LPH |
| 75 | YS098_A | 0.41 | 1.41 | -1.00 | 1.04 | -0.97 | LPH |
| 76 | YS099_A | 0.92 | 2.76 | -1.84 | 2.39 | -0.77 | LPH |
| 77 | YS100_A | 2.34 | 0.72 | 1.62 | 0.35 | 4.65 | HPH |
| 78 | YS101_A | 0.44 | 1.78 | -1.34 | 1.41 | -0.95 | LPH |
| 79 | YS102_A | 2.83 | 0.77 | 2.06 | 0.40 | 5.18 | HPH |
| 80 | YS103_A | 3.96 | 2.01 | 1.95 | 1.64 | 1.19 | HPH |
| 81 | YS105_A | 0.24 | 0.58 | -0.33 | 0.20 | -1.64 | HI |
| 82 | YS107_A | 2.38 | 0.35 | 2.03 | 0.02 | 81.61 | HPH |
| 83 | YS108_A | 3.35 | 2.24 | 1.11 | 1.86 | 0.60 | MPH |
| 84 | YS110_A | 4.22 | 1.77 | 2.45 | 1.40 | 1.75 | HPH |
| 85 | YS112_A | 2.64 | 0.21 | 2.43 | 0.16 | 14.85 | HPH |
| 86 | YS113_A | 3.95 | 0.87 | 3.08 | 0.50 | 6.21 | HPH |
| 87 | YS114_A | 1.70 | 2.08 | -0.38 | 1.70 | -0.22 | LPH |
| 88 | YS115_A | 2.20 | 0.49 | 1.70 | 0.12 | 14.33 | HPH |
| 89 | YS116_A | 3.84 | 1.46 | 2.38 | 1.09 | 2.18 | HPH |
| 90 | YS117_A | 0.38 | 2.04 | -1.65 | 1.66 | -0.99 | LPH |
| 91 | YS118_A | 0.06 | 1.04 | -0.98 | 0.67 | -1.46 | HI |
| 92 | YS122_A | 2.77 | 0.92 | 1.85 | 0.54 | 3.41 | HPH |
| 93 | YS123_A | 3.26 | 0.97 | 2.29 | 0.60 | 3.82 | HPH |
| 94 | YS125_A | 1.24 | 2.04 | -0.81 | 1.67 | -0.48 | LPH |
| 95 | YS126_A | 5.61 | 3.07 | 2.53 | 2.70 | 0.94 | MPH |
| 96 | YS128_A | 1.62 | 0.67 | 0.94 | 0.30 | 3.15 | HPH |
| 97 | YS129_A | 2.05 | 0.70 | 1.35 | 0.33 | 4.13 | HPH |
| 98 | YS130_A | 0.38 | 0.58 | -0.19 | 0.20 | -0.94 | LPH |
| 99 | YS132_A | 0.83 | 1.33 | -0.51 | 0.96 | -0.53 | LPH |
| 100 | YS133_A | 2.12 | 0.15 | 1.97 | 0.22 | 8.98 | HPH |
| 101 | YS134_A | 0.62 | 1.00 | -0.38 | 0.63 | -0.61 | LPH |
| 102 | YS135_A | 0.15 | 0.29 | -0.15 | 0.08 | -1.85 | HI |
| 103 | YS137_A | 0.13 | 0.29 | -0.16 | 0.09 | -1.88 | HI |
| 104 | YS138_A | 0.18 | 0.18 | 0.00 | 0.20 | 0.02 | MPH |
| 105 | YS139_A | 0.05 | 0.22 | -0.17 | 0.15 | -1.08 | HI |
| 106 | YS140_A | 2.55 | 0.41 | 2.13 | 0.04 | 54.59 | HPH |
| 107 | YS141_A | 1.06 | 1.89 | -0.83 | 1.52 | -0.55 | LPH |
| 108 | YS143_A | 0.11 | 0.19 | -0.08 | 0.18 | -0.42 | LPH |
| 109 | YS144_A | 2.07 | 0.24 | 1.83 | 0.13 | 13.95 | HPH |
| 110 | YS146_A | 3.48 | 2.29 | 1.19 | 1.92 | 0.62 | MPH |
| 111 | YS147_A | 2.93 | 0.98 | 1.95 | 0.61 | 3.21 | HPH |
| 112 | YS148_A | 0.29 | 0.47 | -0.18 | 0.09 | -1.93 | HI |
| 113 | YS149_A | 1.77 | 0.57 | 1.20 | 0.20 | 6.07 | HPH |
| 114 | YS151_A | 0.47 | 1.33 | -0.85 | 0.95 | -0.90 | LPH |
| 115 | YS152_A | 4.26 | 2.32 | 1.94 | 1.95 | 0.99 | MPH |
| 116 | YS153_A | 3.26 | 1.13 | 2.13 | 0.76 | 2.81 | HPH |
| 117 | YS154_A | 4.23 | 1.32 | 2.91 | 0.95 | 3.07 | HPH |
| 118 | YS155_A | 2.50 | 0.43 | 2.07 | 0.05 | 38.62 | HPH |
| 119 | YS156_A | 3.97 | 2.26 | 1.72 | 1.88 | 0.91 | MPH |
| 120 | YS158_A | 3.97 | 0.46 | 3.51 | 0.09 | 38.49 | HPH |
| 121 | YS159_A | 3.15 | 0.30 | 2.85 | 0.07 | 41.11 | HPH |
| 122 | YS160_A | 3.80 | 1.74 | 2.05 | 1.37 | 1.50 | HPH |
| 123 | YS161_A | 4.36 | 2.17 | 2.19 | 1.79 | 1.22 | HPH |
| 124 | YS163_A | 4.08 | 1.95 | 2.13 | 1.57 | 1.36 | HPH |
| 125 | YS164_A | 4.78 | 1.96 | 2.82 | 1.59 | 1.77 | HPH |
| 126 | YS165_A | 4.82 | 2.24 | 2.57 | 1.87 | 1.37 | HPH |
| 127 | YS166_A | 4.40 | 2.42 | 1.98 | 2.05 | 0.97 | MPH |
| 128 | YS167_A | 3.65 | 0.42 | 3.23 | 0.05 | 71.22 | HPH |
| 129 | YS168_A | 0.52 | 0.73 | -0.22 | 0.36 | -0.60 | LPH |
| 130 | YS169_A | 0.30 | 0.68 | -0.38 | 0.31 | -1.24 | HI |
| 131 | YS170_A | 0.92 | 1.36 | -0.44 | 0.99 | -0.45 | LPH |
| 132 | YS171_A | 0.57 | 0.43 | 0.14 | 0.06 | 2.53 | HPH |
| 133 | YS172_A | 2.68 | 1.19 | 1.49 | 0.81 | 1.84 | HPH |
| 134 | YS173_A | 4.26 | 2.32 | 1.94 | 1.95 | 1.00 | MPH |
| 135 | YS175_A | 3.72 | 1.76 | 1.96 | 1.39 | 1.41 | HPH |
| 136 | YS177_A | 4.72 | 1.95 | 2.77 | 1.57 | 1.76 | HPH |
| 137 | YS178_A | 3.52 | 1.27 | 2.25 | 0.90 | 2.50 | HPH |
| 138 | YS180_A | 4.82 | 2.59 | 2.22 | 2.22 | 1.00 | MPH |
| 139 | YS181_A | 4.24 | 2.35 | 1.89 | 1.97 | 0.96 | MPH |
| 140 | YS183_A | 4.70 | 2.31 | 2.39 | 1.94 | 1.23 | HPH |
| 141 | YS184_A | 1.83 | 1.18 | 0.65 | 0.81 | 0.80 | MPH |
| 142 | YS185_A | 4.65 | 1.91 | 2.74 | 1.54 | 1.78 | HPH |
| 143 | YS186_A | 5.16 | 2.83 | 2.33 | 2.46 | 0.95 | MPH |
| 144 | YS187_A | 0.09 | 1.16 | -1.07 | 0.78 | -1.36 | HI |
| 145 | YS189_A | 0.98 | 0.46 | 0.53 | 0.08 | 6.25 | HPH |
| 146 | YS190_A | 1.28 | 0.70 | 0.58 | 0.32 | 1.80 | HPH |
| 147 | YS191_A | 1.71 | 2.20 | -0.49 | 1.83 | -0.27 | LPH |
| 148 | YS192_A | 0.21 | 0.87 | -0.66 | 0.50 | -1.32 | HI |
| 149 | YS193_A | 3.65 | 0.23 | 3.42 | 0.14 | 24.50 | HPH |
| 150 | YS194_A | 0.36 | 0.45 | -0.09 | 0.07 | -1.16 | HI |
| 151 | YS195_A | 2.52 | 0.77 | 1.75 | 0.39 | 4.44 | HPH |
| 152 | YS196_A | 4.33 | 1.69 | 2.64 | 1.32 | 2.00 | HPH |
| 153 | YS198_A | 1.33 | 0.88 | 0.45 | 0.50 | 0.90 | MPH |
| 154 | YS199_A | 3.90 | 1.76 | 2.15 | 1.38 | 1.55 | HPH |
| 155 | YS200_A | 0.32 | 0.34 | -0.02 | 0.03 | -0.75 | LPH |
| 156 | YS201_A | 4.64 | 2.08 | 2.56 | 1.71 | 1.50 | HPH |
| 157 | YS202_A | 4.40 | 2.19 | 2.21 | 1.82 | 1.22 | HPH |
| 158 | YS203_A | 0.06 | 0.26 | -0.19 | 0.12 | -1.66 | HI |
| 159 | YS204_A | 2.73 | 1.29 | 1.43 | 0.92 | 1.56 | HPH |
| 160 | YS205_A | 0.45 | 0.36 | 0.09 | 0.02 | 5.60 | HPH |
| 161 | YS206_A | 2.08 | 2.27 | -0.19 | 1.90 | -0.10 | LPH |
| 162 | YS207_A | 0.35 | 0.87 | -0.52 | 0.50 | -1.05 | HI |
| 163 | YS208_A | 0.61 | 1.65 | -1.04 | 1.28 | -0.81 | LPH |
| 164 | YS209_A | 0.41 | 0.82 | -0.41 | 0.45 | -0.91 | LPH |
| 165 | YS210_A | 0.50 | 1.85 | -1.34 | 1.47 | -0.91 | LPH |
| 166 | YS211_A | 0.08 | 0.29 | -0.21 | 0.08 | -2.58 | HI |
| 167 | YS212_A | 4.28 | 2.54 | 1.74 | 2.17 | 0.80 | MPH |
| 168 | YS213_A | 0.72 | 1.18 | -0.46 | 0.80 | -0.57 | LPH |
| 169 | YS214_A | 0.45 | 1.48 | -1.03 | 1.10 | -0.93 | LPH |
| 170 | YS215_A | 2.16 | 0.76 | 1.39 | 0.39 | 3.57 | HPH |
| 171 | YS216_A | 0.17 | 0.83 | -0.65 | 0.45 | -1.44 | HI |
| 172 | YS217_A | 0.47 | 0.43 | 0.04 | 0.06 | 0.63 | MPH |
| 173 | YS218_A | 0.23 | 0.63 | -0.39 | 0.25 | -1.55 | HI |
| 174 | YS219_A | 0.26 | 0.76 | -0.50 | 0.39 | -1.29 | HI |
| 175 | YS220_A | 2.34 | 1.00 | 1.35 | 0.62 | 2.16 | HPH |
| 176 | YS221_A | 0.27 | 1.48 | -1.22 | 1.11 | -1.10 | HI |
| 177 | YS222_A | 3.74 | 1.92 | 1.83 | 1.54 | 1.18 | HPH |
| 178 | YS223_A | 4.54 | 2.60 | 1.93 | 2.23 | 0.87 | MPH |
| 179 | YS226_A | 0.19 | 1.07 | -0.89 | 0.70 | -1.27 | HI |
| 180 | YS227_A | 0.53 | 0.80 | -0.27 | 0.43 | -0.62 | LPH |
| 181 | YS228_A | 0.20 | 0.40 | -0.20 | 0.03 | -7.37 | HI |
| 182 | YS229_A | 0.21 | 0.74 | -0.53 | 0.37 | -1.43 | HI |
| 183 | YS230_A | 2.66 | 0.38 | 2.28 | 0.01 | 316.52 | HPH |
| 184 | YS231_A | 4.32 | 2.40 | 1.92 | 2.03 | 0.94 | MPH |
| 185 | YS232_A | 0.33 | 1.85 | -1.52 | 1.48 | -1.03 | HI |
| 186 | YS233_A | 0.27 | 1.67 | -1.40 | 1.30 | -1.08 | HI |
| 187 | YS234_A | 0.66 | 1.51 | -0.85 | 1.14 | -0.75 | LPH |
| 188 | YS235_A | 0.49 | 1.81 | -1.32 | 1.44 | -0.92 | LPH |
| 189 | YS236_A | 0.13 | 1.02 | -0.88 | 0.64 | -1.37 | HI |
| 190 | YS238_A | 0.34 | 1.42 | -1.07 | 1.05 | -1.03 | HI |
| 191 | YS239_A | 4.69 | 0.63 | 4.06 | 0.26 | 15.91 | HPH |
| 192 | YS240_A | 0.34 | 1.47 | -1.13 | 1.09 | -1.03 | HI |
| 193 | YS241_A | 0.36 | 1.98 | -1.62 | 1.60 | -1.01 | HI |
| 194 | YS242_A | 2.01 | 2.39 | -0.38 | 2.02 | -0.19 | LPH |
| 195 | YS243_A | 0.49 | 1.43 | -0.94 | 1.06 | -0.89 | LPH |
| 196 | YS244_A | 0.16 | 1.05 | -0.89 | 0.68 | -1.32 | HI |
| 197 | YS245_A | 3.09 | 0.91 | 2.19 | 0.53 | 4.11 | HPH |
| 198 | YS246_A | 2.79 | 0.20 | 2.59 | 0.17 | 14.83 | HPH |
| 199 | YS248_A | 0.59 | 1.51 | -0.92 | 1.14 | -0.81 | LPH |
| 200 | YS250_A | 0.10 | 0.15 | -0.05 | 0.22 | -0.24 | LPH |
| 201 | YS251_A | 0.12 | 0.28 | -0.16 | 0.09 | -1.75 | HI |
| 202 | YS252_A | 0.49 | 0.72 | -0.23 | 0.35 | -0.65 | LPH |
| 203 | YS253_A | 4.25 | 1.87 | 2.37 | 1.50 | 1.59 | HPH |
| 204 | YS254_A | 2.75 | 0.34 | 2.41 | 0.03 | 82.81 | HPH |
| 205 | YS255_A | 1.03 | 2.29 | -1.27 | 1.92 | -0.66 | LPH |
| 206 | YS256_A | 2.42 | 1.28 | 1.14 | 0.91 | 1.26 | HPH |
| 207 | YS257_A | 1.76 | 2.94 | -1.18 | 2.56 | -0.46 | LPH |
| 208 | YS259_A | 5.07 | 2.37 | 2.70 | 1.99 | 1.36 | HPH |
| 209 | YS260_A | 2.78 | 2.81 | -0.03 | 2.44 | -0.01 | LPH |
| 210 | YS263_A | 4.70 | 2.36 | 2.34 | 1.99 | 1.17 | HPH |
| 211 | YS264_A | 3.76 | 1.81 | 1.95 | 1.43 | 1.36 | HPH |
| 212 | YS265_A | 5.37 | 2.64 | 2.73 | 2.27 | 1.20 | HPH |
| 213 | YS266_A | 2.21 | 0.42 | 1.79 | 0.04 | 41.12 | HPH |
| 214 | YS267_A | 4.61 | 2.22 | 2.39 | 1.85 | 1.29 | HPH |
| 215 | YS268_A | 3.65 | 1.14 | 2.52 | 0.76 | 3.30 | HPH |
| 216 | YS269_A | 4.50 | 2.18 | 2.32 | 1.81 | 1.28 | HPH |
| 217 | YS270_A | 4.78 | 2.47 | 2.31 | 2.10 | 1.10 | HPH |
| 218 | YS271_A | 0.47 | 2.31 | -1.84 | 1.93 | -0.95 | LPH |
| 219 | YS272_A | 5.18 | 1.98 | 3.20 | 1.61 | 1.99 | HPH |
| 220 | YS273_A | 0.39 | 0.40 | -0.01 | 0.03 | -0.49 | LPH |
| 221 | YS275_A | 5.16 | 2.64 | 2.51 | 2.27 | 1.11 | HPH |
| 222 | YS279_A | 0.56 | 0.73 | -0.17 | 0.35 | -0.48 | LPH |
| 223 | YS280_A | 4.26 | 1.33 | 2.93 | 0.96 | 3.06 | HPH |
| 224 | YS281_A | 0.10 | 0.63 | -0.54 | 0.26 | -2.06 | HI |
| 225 | YS282_A | 5.41 | 3.03 | 2.38 | 2.66 | 0.89 | MPH |
| 226 | YS283_A | 2.71 | 2.19 | 0.51 | 1.82 | 0.28 | MPH |
| 227 | YS284_A | 1.50 | 2.07 | -0.57 | 1.70 | -0.34 | LPH |
| 228 | YS285_A | 3.69 | 1.33 | 2.36 | 0.96 | 2.45 | HPH |
| 229 | YS286_A | 0.53 | 1.71 | -1.18 | 1.33 | -0.88 | LPH |
| 230 | YS288_A | 0.25 | 0.13 | 0.12 | 0.24 | 0.49 | MPH |
| 231 | YS289_A | 1.81 | 0.34 | 1.47 | 0.03 | 51.12 | HPH |
| 232 | YS292_A | 0.39 | 1.87 | -1.47 | 1.49 | -0.99 | LPH |
| 233 | YS295_A | 0.40 | 1.51 | -1.11 | 1.13 | -0.98 | LPH |
| 234 | YS298_A | 0.44 | 1.31 | -0.88 | 0.94 | -0.93 | LPH |
| 235 | YS299_A | 0.72 | 1.86 | -1.14 | 1.48 | -0.77 | LPH |
| 236 | YS302_A | 0.02 | 0.34 | -0.32 | 0.03 | -10.69 | HI |
| 237 | YS303_A | 0.63 | 1.03 | -0.40 | 0.65 | -0.61 | LPH |
| 238 | YS304_A | 0.78 | 2.68 | -1.90 | 2.31 | -0.82 | LPH |
| 239 | YS309_A | 0.08 | 0.64 | -0.56 | 0.27 | -2.08 | HI |
| 240 | YS315_A | 1.37 | 2.42 | -1.05 | 2.05 | -0.51 | LPH |
| 241 | YS317_A | 4.62 | 2.52 | 2.11 | 2.14 | 0.98 | MPH |
| 242 | YS318_A | 4.91 | 2.62 | 2.28 | 2.25 | 1.02 | HPH |
| 243 | YS319_A | 3.95 | 2.39 | 1.56 | 2.02 | 0.77 | MPH |
| 244 | YS324_A | 4.63 | 2.34 | 2.29 | 1.97 | 1.16 | HPH |
| 245 | YS325_A | 0.98 | 0.63 | 0.35 | 0.26 | 1.34 | HPH |
| 246 | YS327_A | 3.72 | 1.29 | 2.43 | 0.91 | 2.66 | HPH |
| 247 | YS331_A | 4.37 | 2.30 | 2.07 | 1.92 | 1.08 | HPH |
| 248 | YS332_A | 4.50 | 2.04 | 2.47 | 1.66 | 1.48 | HPH |
| 249 | YS334_A | 0.97 | 1.24 | -0.27 | 0.86 | -0.31 | LPH |
| 250 | YS340_A | 3.11 | 0.28 | 2.83 | 0.09 | 30.83 | HPH |
| 251 | YS341_A | 3.61 | 0.70 | 2.91 | 0.33 | 8.91 | HPH |
| 252 | YS343_A | 1.08 | 0.77 | 0.31 | 0.40 | 0.78 | MPH |
| 253 | YS345_A | 3.51 | 0.88 | 2.63 | 0.51 | 5.19 | HPH |
| 254 | YS349_A | 3.62 | 2.30 | 1.32 | 1.93 | 0.68 | MPH |
| 255 | YS350_A | 4.50 | 2.21 | 2.29 | 1.84 | 1.25 | HPH |
| 256 | YS351_A | 2.00 | 0.32 | 1.69 | 0.06 | 29.50 | HPH |
| 257 | YS353_A | 4.45 | 2.54 | 1.90 | 2.17 | 0.88 | MPH |
| 258 | YS355_A | 3.47 | 1.47 | 2.00 | 1.10 | 1.83 | HPH |
| 259 | YS356_A | 1.24 | 0.70 | 0.54 | 0.33 | 1.65 | HPH |
| 260 | YS358_A | 5.12 | 1.94 | 3.17 | 1.57 | 2.02 | HPH |
| 261 | YS359_A | 4.69 | 2.50 | 2.19 | 2.12 | 1.03 | HPH |
| 262 | YS360_A | 1.76 | 0.21 | 1.56 | 0.17 | 9.40 | HPH |
| 263 | YS363_A | 4.27 | 2.10 | 2.18 | 1.72 | 1.26 | HPH |
| 264 | YS364_A | 4.99 | 2.12 | 2.87 | 1.75 | 1.65 | HPH |
| 265 | YS365_A | 4.37 | 1.87 | 2.50 | 1.49 | 1.68 | HPH |
| 266 | YS368_A | 4.38 | 2.53 | 1.85 | 2.16 | 0.86 | MPH |
| 267 | YS372_A | 4.71 | 2.27 | 2.44 | 1.89 | 1.29 | HPH |
| 268 | YS374_A | 1.93 | 1.18 | 0.75 | 0.80 | 0.94 | MPH |
| 269 | YS375_A | 3.02 | 1.77 | 1.25 | 1.40 | 0.89 | MPH |
| 270 | YS376_A | 3.73 | 2.17 | 1.56 | 1.80 | 0.87 | MPH |
| 271 | YS377_A | 0.49 | 2.71 | -2.21 | 2.33 | -0.95 | LPH |
| 272 | YS378_A | 1.36 | 2.03 | -0.68 | 1.66 | -0.41 | LPH |
| 273 | YS379_A | 1.23 | 1.83 | -0.60 | 1.46 | -0.41 | LPH |
| 274 | YS380_A | 3.13 | 1.50 | 1.63 | 1.13 | 1.45 | HPH |
| 275 | YS381_A | 4.37 | 2.29 | 2.08 | 1.91 | 1.09 | HPH |
| 276 | YS382_A | 1.71 | 2.21 | -0.50 | 1.83 | -0.27 | LPH |
| 277 | YS384_A | 5.28 | 2.84 | 2.44 | 2.47 | 0.99 | MPH |
| 278 | YS385_A | 3.84 | 2.19 | 1.64 | 1.82 | 0.90 | MPH |
| 279 | YS386_A | 0.62 | 1.78 | -1.16 | 1.41 | -0.82 | LPH |
| 280 | YS387_A | 1.98 | 2.30 | -0.32 | 1.92 | -0.17 | LPH |
| 281 | YS388_A | 1.32 | 2.36 | -1.04 | 1.99 | -0.52 | LPH |
| 282 | YS390_A | 1.66 | 2.75 | -1.10 | 2.38 | -0.46 | LPH |
| 283 | YS392_A | 1.34 | 2.45 | -1.11 | 2.08 | -0.54 | LPH |
| 284 | YS394_A | 2.62 | 1.80 | 0.82 | 1.43 | 0.57 | MPH |
| 285 | YS399_A | 0.75 | 2.03 | -1.28 | 1.65 | -0.77 | LPH |
| 286 | YS400_A | 4.32 | 2.38 | 1.94 | 2.00 | 0.97 | MPH |
| 287 | YS401_A | 4.31 | 2.20 | 2.11 | 1.83 | 1.15 | HPH |
| 288 | YS402_A | 4.78 | 2.46 | 2.32 | 2.09 | 1.11 | HPH |
| 289 | YS405_A | 1.70 | 1.80 | -0.10 | 1.43 | -0.07 | LPH |
| 290 | YS607_A | 2.33 | 0.38 | 1.96 | 0.00 | 528.37 | HPH |
| 291 | YS608_A | 3.05 | 0.75 | 2.30 | 0.38 | 6.06 | HPH |
| 292 | YS610_A | -0.06 | 0.14 | -0.20 | 0.23 | -0.88 | LPH |
| 293 | YS611_A | 0.46 | 1.00 | -0.54 | 0.63 | -0.86 | LPH |
| 294 | YS612_A | 0.22 | 0.22 | 0.00 | 0.16 | 0.03 | MPH |
| 295 | YS613_A | 2.48 | 0.53 | 1.95 | 0.16 | 12.46 | HPH |
| 296 | YS614_A | 0.19 | 0.30 | -0.11 | 0.07 | -1.45 | HI |
| 297 | YS615_A | 0.20 | 0.25 | -0.05 | 0.12 | -0.43 | LPH |
| 298 | YS616_A | 0.26 | 0.27 | -0.01 | 0.10 | -0.13 | LPH |
| 299 | YS617_A | 2.67 | 0.74 | 1.94 | 0.37 | 5.30 | HPH |
| 300 | YS618_A | 2.50 | 0.55 | 1.95 | 0.18 | 11.07 | HPH |
| 301 | YS619_A | 4.01 | 2.51 | 1.50 | 2.14 | 0.70 | MPH |
| 302 | YS620_A | 0.64 | 0.18 | 0.46 | 0.20 | 2.34 | HPH |
| 303 | YS621_A | 0.18 | 0.24 | -0.05 | 0.14 | -0.40 | LPH |
| 304 | YS622_A | 1.41 | 2.05 | -0.63 | 1.67 | -0.38 | LPH |
| 305 | YS623_A | 0.07 | 0.25 | -0.18 | 0.12 | -1.46 | HI |
| 306 | YS624_A | 0.32 | 0.32 | 0.00 | 0.05 | -0.03 | LPH |
| 307 | YS625_A | 0.28 | 1.00 | -0.73 | 0.63 | -1.15 | HI |
| 308 | YS626_A | 0.01 | 1.40 | -1.39 | 1.03 | -1.35 | HI |
| 309 | YS627_A | 0.19 | 0.36 | -0.17 | 0.01 | -20.86 | HI |
| 310 | YS628_A | 0.25 | 0.25 | 0.00 | 0.12 | 0.02 | MPH |
| 311 | YS629_A | -0.04 | 0.14 | -0.18 | 0.23 | -0.77 | LPH |
| 312 | YS630_A | 1.28 | 0.21 | 1.07 | 0.17 | 6.40 | HPH |
| 313 | YS631_A | 0.23 | 0.40 | -0.18 | 0.03 | -5.97 | HI |
| 314 | YS632_A | 0.23 | 0.40 | -0.17 | 0.02 | -6.92 | HI |
| 315 | YS633_A | -0.10 | 0.17 | -0.27 | 0.20 | -1.30 | HI |
| 316 | YS634_A | -0.05 | 0.19 | -0.23 | 0.18 | -1.26 | HI |
| 317 | YS635_A | -0.06 | 0.28 | -0.34 | 0.09 | -3.79 | HI |
| 318 | YS636_A | 0.06 | 0.20 | -0.14 | 0.18 | -0.79 | LPH |
| 319 | YS637_A | 0.03 | 0.25 | -0.22 | 0.12 | -1.81 | HI |
| 320 | YS638_A | -0.02 | 0.27 | -0.29 | 0.10 | -2.91 | HI |
| 321 | YS639_A | 0.15 | 0.16 | -0.02 | 0.21 | -0.08 | LPH |
| 322 | YS640_A | 0.21 | 0.34 | -0.13 | 0.03 | -4.48 | HI |
| 323 | YS641_A | 0.48 | 0.14 | 0.34 | 0.23 | 1.45 | HPH |
| 324 | YS642_A | 0.15 | 1.33 | -1.17 | 0.95 | -1.23 | HI |
| 325 | YS643_A | 0.29 | 0.88 | -0.59 | 0.51 | -1.16 | HI |
| 326 | YS645_A | 2.98 | 1.26 | 1.72 | 0.89 | 1.93 | HPH |
| 327 | YS646_A | 0.12 | 0.24 | -0.12 | 0.14 | -0.85 | LPH |
| 328 | YS647_A | 0.79 | 2.15 | -1.35 | 1.77 | -0.76 | LPH |
| 329 | YS648_A | 0.13 | 0.14 | -0.01 | 0.23 | -0.05 | LPH |
| 330 | YS649_A | 2.74 | 0.19 | 2.55 | 0.18 | 14.07 | HPH |
| 331 | YS650_A | 0.12 | 0.15 | -0.03 | 0.23 | -0.13 | LPH |
| 332 | YS651_A | 0.49 | 1.09 | -0.60 | 0.72 | -0.83 | LPH |
| 333 | YS652_A | 0.47 | 0.72 | -0.25 | 0.35 | -0.72 | LPH |
| 334 | YS653_A | 0.25 | 0.32 | -0.08 | 0.05 | -1.50 | HI |
| 335 | YS654_A | 0.25 | 0.49 | -0.24 | 0.12 | -1.99 | HI |
| 336 | YS655_A | 0.60 | 0.35 | 0.24 | 0.02 | 12.98 | HPH |
| 337 | YS656_A | 0.24 | 0.49 | -0.25 | 0.12 | -2.12 | HI |
| 338 | YS658_A | 0.39 | 0.30 | 0.09 | 0.07 | 1.28 | HPH |
| 339 | YS659_A | 0.32 | 1.44 | -1.12 | 1.07 | -1.05 | HI |
| 340 | YS660_A | 0.22 | 0.56 | -0.34 | 0.19 | -1.81 | HI |
| 341 | YS661_A | 0.10 | 0.32 | -0.21 | 0.06 | -3.76 | HI |
| 342 | YS662_A | 0.32 | 0.17 | 0.15 | 0.20 | 0.75 | MPH |
| 343 | YS663_A | 0.35 | 0.50 | -0.15 | 0.12 | -1.19 | HI |
| 344 | YS664_A | 3.29 | 0.45 | 2.84 | 0.08 | 37.36 | HPH |
| 345 | YS665_A | 4.34 | 2.39 | 1.95 | 2.02 | 0.97 | MPH |
| 346 | YS667_A | 2.68 | 1.05 | 1.63 | 0.68 | 2.40 | HPH |
| 347 | YS669_A | 3.26 | 0.80 | 2.46 | 0.43 | 5.70 | HPH |
| 348 | YS670_A | 4.29 | 2.07 | 2.22 | 1.69 | 1.31 | HPH |
| 349 | YS671_A | 4.25 | 1.60 | 2.65 | 1.23 | 2.16 | HPH |
| 350 | YS672_A | 3.03 | 1.24 | 1.79 | 0.87 | 2.05 | HPH |
| 351 | YS673_A | 0.36 | 0.70 | -0.34 | 0.33 | -1.04 | HI |
| 352 | YS676_A | 2.78 | 0.38 | 2.40 | 0.01 | 316.56 | HPH |
| 353 | YS677_A | 0.88 | 1.91 | -1.03 | 1.54 | -0.67 | LPH |
| 354 | YS678_A | 3.66 | 1.91 | 1.74 | 1.54 | 1.13 | HPH |
| 355 | YS679_A | 2.68 | 0.27 | 2.41 | 0.10 | 23.21 | HPH |
| 356 | YS680_A | 0.92 | 1.33 | -0.40 | 0.95 | -0.43 | LPH |
| 357 | YS681_A | 3.92 | 1.34 | 2.58 | 0.97 | 2.66 | HPH |
| 358 | YS682_A | 2.74 | 0.19 | 2.55 | 0.19 | 13.77 | HPH |
| 359 | YS683_A | 4.41 | 0.88 | 3.54 | 0.50 | 7.04 | HPH |
| 360 | YS684_A | 3.36 | 0.29 | 3.07 | 0.08 | 37.85 | HPH |
| 361 | YS685_A | 5.37 | 2.56 | 2.81 | 2.19 | 1.28 | HPH |
| 362 | YS686_A | 3.85 | 0.37 | 3.48 | 0.00 | 965.68 | HPH |
| 363 | YS687_A | 0.17 | 0.12 | 0.05 | 0.26 | 0.19 | MPH |
| 364 | YS688_A | 0.24 | 0.23 | 0.01 | 0.14 | 0.06 | MPH |
| 365 | YS690_A | 3.77 | 0.78 | 2.99 | 0.40 | 7.44 | HPH |
| 366 | YS691_A | 3.83 | 1.46 | 2.37 | 1.09 | 2.18 | HPH |
| 367 | YS692_A | 0.96 | 2.59 | -1.63 | 2.21 | -0.74 | LPH |
| 368 | YS693_A | 1.62 | 0.14 | 1.48 | 0.23 | 6.31 | HPH |
| 369 | YS694_A | 0.29 | 0.48 | -0.19 | 0.10 | -1.84 | HI |
| 370 | YS695_A | 1.11 | 0.58 | 0.53 | 0.21 | 2.56 | HPH |
| 371 | YS696_A | 4.14 | 1.17 | 2.97 | 0.80 | 3.70 | HPH |
| 372 | YS697_A | 3.37 | 0.57 | 2.80 | 0.20 | 14.04 | HPH |
| 373 | YS698_A | 0.41 | 0.25 | 0.16 | 0.13 | 1.32 | HPH |
| 374 | YS700_A | 1.80 | 0.19 | 1.62 | 0.19 | 8.72 | HPH |
| 375 | YS701_A | 3.43 | 0.62 | 2.81 | 0.25 | 11.39 | HPH |
| 376 | YS702_A | 0.04 | 0.16 | -0.11 | 0.22 | -0.51 | LPH |
| 377 | YS705_A | 3.20 | 0.19 | 3.01 | 0.19 | 16.21 | HPH |
| 378 | YS706_A | 0.40 | 0.36 | 0.04 | 0.02 | 2.42 | HPH |
| 379 | YS707_A | 4.35 | 2.37 | 1.98 | 1.99 | 1.00 | MPH |
| 380 | YS708_A | 4.12 | 1.23 | 2.89 | 0.86 | 3.35 | HPH |
| 381 | YS709_A | 3.89 | 2.27 | 1.62 | 1.90 | 0.85 | MPH |
| 382 | YS710_A | 4.81 | 2.59 | 2.22 | 2.22 | 1.00 | MPH |
| 383 | YS711_A | 0.62 | 1.42 | -0.79 | 1.04 | -0.76 | LPH |
| 384 | YS712_A | 2.99 | 0.33 | 2.66 | 0.04 | 64.26 | HPH |
| 385 | YS713_A | 0.77 | 0.34 | 0.42 | 0.03 | 14.48 | HPH |
| 386 | YS714_A | 0.98 | 1.79 | -0.80 | 1.41 | -0.57 | LPH |
| 387 | YS715_A | 0.80 | 1.28 | -0.48 | 0.91 | -0.52 | LPH |
| 388 | YS716_A | 1.07 | 0.33 | 0.74 | 0.04 | 16.96 | HPH |
| 389 | YS719_A | 0.44 | 0.31 | 0.13 | 0.06 | 2.03 | HPH |
| 390 | YS720_A | 0.42 | 0.30 | 0.11 | 0.07 | 1.65 | HPH |
| 391 | YS726_A | 1.91 | 1.89 | 0.02 | 1.51 | 0.01 | MPH |
| 392 | YS728_A | 0.87 | 1.93 | -1.06 | 1.56 | -0.68 | LPH |
| 393 | YS737_A | 0.69 | 1.59 | -0.89 | 1.21 | -0.74 | LPH |
| 394 | YS754_A | 0.51 | 2.19 | -1.68 | 1.82 | -0.92 | LPH |
| 395 | YS758_A | 0.64 | 0.96 | -0.32 | 0.58 | -0.55 | LPH |
| 396 | YS765_A | 0.57 | 1.87 | -1.30 | 1.50 | -0.87 | LPH |
| 397 | YS783_A | 1.75 | 1.94 | -0.18 | 1.56 | -0.12 | LPH |
| 398 | YS790_A | 3.02 | 1.39 | 1.63 | 1.02 | 1.60 | HPH |
| 399 | YS803_A | 0.76 | 1.52 | -0.76 | 1.14 | -0.66 | LPH |
| 400 | YS805_A | 3.23 | 1.90 | 1.33 | 1.53 | 0.87 | MPH |
| 401 | YS820_A | 4.41 | 2.34 | 2.07 | 1.96 | 1.05 | HPH |

Mid-parent value is the average best linear unbiased estimates (BLUE) of mesocotyl length of parents of the hybrids. d-value is the difference of BLUE between hybrid and its mid-parent value. a-value is absolute value of the difference of BLUE between parents. Hp is d-value / a-value. High parent heterosis (HPH) represents the Hp of hybrid was over 1. Mid-parent heterosis (MPH) represents the Hp of hybrid was over 0 and below 1 (including 1). Low-parent heterosis (LPH) represents the Hp of hybrid was over -1(including -1) and below 0. Hybrid inferiority (HI) represents the Hp of hybrid was below -1.
