# Supplementary material for: Genomic prediction of rice mesocotyl length indicative of directing seeding suitability using a half-sib hybrid population
Source: PLoS One. 2023 Apr 5;18(4):e0283989. doi: 10.1371/journal.pone.0283989 (PMC10075464; doi:10.1371/journal.pone.0283989)
Supplement: S5 Table — The asterisks indicate the Fisher’s z-transformed prediction accuracies in scenarios 1–5 were significantly higher (p < 0.05, t-test) than the Fisher’s z-transformed mid-parental value prediction accuracy. (DOCX) [file pone.0283989.s007.docx]

**Supplementary Table S5.** The prediction accuracies of scenario 1-5 in MAS.

| **Scenario** | **P=5e-5** | **P=1e-4** | **P=5e-4** | **P=1e-3** | **P=2.5e-3** | **P=5e-3** | **P=7.5e-3** | **P=0.01** |
| --- | --- | --- | --- | --- | --- | --- | --- | --- |
| **1: Reference hybrids’ parents** | 0.26±0.04 | 0.28±0.03 | 0.37±0.04 | **0.39±0.03** | 0.37±0.05 | 0.15±0.08 | 0.02±0.05 | 0±0.06 |
| **2: Reference hybrids** | 0.10±0.04 | 0.13±0.04 | 0.32±0.03 | 0.40±0.03 | **0.48±0.03** | 0.39±0.06 | 0.10±0.08 | 0.00±0.04 |
| **3: Reference hybrids and their parents** | 0.31±0.03 | 0.34±0.03 | 0.48±0.02 | **0.51±0.02** | 0.41±0.06 | 0.19±0.07 | 0.01±0.05 | 0.01±0.04 |
| **4: Reference hybrids and all lines** | 0.39±0.02 | 0.42±0.01 | 0.54±0.01 | 0.59±0.01 | 0.60±0.01^*^ | **0.61±0.01^*^** | 0.59±0.00^*^ | 0.54±0.16 |
| **5: Reference hybrids and parents of test set** | 0.18±0.06 | 0.23±0.05 | 0.43±0.03 | 0.51±0.03 | 0.59±0.02 | **0.61±0.01^*^** | 0.60±0.01^*^ | 0.59±0.01 |

The asterisks indicate the prediction accuracies were significantly higher (*p* < 0.05, *t*-test) than the mid-parental value prediction accuracy.
