# Supplementary material for: Genomic prediction of rice mesocotyl length indicative of directing seeding suitability using a half-sib hybrid population
Source: PLoS One. 2023 Apr 5;18(4):e0283989. doi: 10.1371/journal.pone.0283989 (PMC10075464; doi:10.1371/journal.pone.0283989)
Supplement: S6 Table — Scenario and P value are the two factors. Scenario:P value represents the interaction effect between scenario and P value. Df represents degree of freedom. SS represents sum of squares. MS represents mean squares. F value is MS / MSError. P (F) is the P value of F-test. All prediction accuracies were Fisher’s z-transformed. (DOCX) [file pone.0283989.s008.docx]

**Supplementary Table S6.** Two-Way ANOVA in MAS prediction accuracies.

| **Factor** | **Df** | **SS** | **MS** | **F value** | **P (F)** |
| --- | --- | --- | --- | --- | --- |
| **Scenario** | 4 | 18.336506 | 4.584127 | 1650.451896 | 0 |
| **P value** | 7 | 10.054143 | 1.436306 | 517.122320 | 4.80E-284 |
| **Scenario:P value** | 28 | 13.450779 | 0.480385 | 172.956019 | 2.19E-307 |
| **Error** | 760 | 2.110898 | 0.002777 |  |  |

Scenario and P value are the two factors. Scenario:P value represents the interaction effect between scenario and P value. Df represents degree of freedom. SS represents sum of squares. MS represents mean squares. F value is MS / MS_Error_. P (F) is the P value of *F*-test.
