# Supplementary material for: Genomic prediction of rice mesocotyl length indicative of directing seeding suitability using a half-sib hybrid population
Source: PLoS One. 2023 Apr 5;18(4):e0283989. doi: 10.1371/journal.pone.0283989 (PMC10075464; doi:10.1371/journal.pone.0283989)
Supplement: S7 Table — Scenario and prediction model are the two factors. Scenario:prediction model represents the interaction effect between scenario and prediction model. Df represents degree of freedom. SS represents sum of squares. MS represents mean squares. F value is MS / MSError. P (F) is the P value of F-test. All prediction accuracies were Fisher’s z-transformed. (DOCX) [file pone.0283989.s009.docx]

**Supplementary Table S7.** Two-Way ANOVA in GS prediction accuracies.

| **Factor** | **Df** | **SS** | **MS** | **F value** | **P (F)** |
| --- | --- | --- | --- | --- | --- |
| **Scenario** | 4 | 1.975250 | 0.493812 | 2949.125534 | 1.50E-284 |
| **Prediction model** | 3 | 0.045178 | 0.015059 | 89.936122 | 5.41E-44 |
| **Scenario:Prediction model** | 12 | 0.046131 | 0.003844 | 22.958682 | 3.10E-38 |
| **Error** | 380 | 0.063629 | 0.000167 |  |  |

Scenario and prediction model are the two factors. Scenario:prediction model represents the interaction effect between scenario and prediction model. Df represents degree of freedom. SS represents sum of squares. MS represents mean squares. F value is MS / MS_Error_. P (F) is the P value of *F*-test.
