# Supplementary material for: Genomic prediction of rice mesocotyl length indicative of directing seeding suitability using a half-sib hybrid population
Source: PLoS One. 2023 Apr 5;18(4):e0283989. doi: 10.1371/journal.pone.0283989 (PMC10075464; doi:10.1371/journal.pone.0283989)
Supplement: S8 Table — (DOCX) [file pone.0283989.s010.docx]

**Supplementary Table S8.** The specific sample size of training set in all scenarios.

| Sample size | Reference hybrids' prarents | Reference hybrids | Reference hybrids and their parents | Reference hybrids and all lines | Reference hybrids and parents of test set |
| --- | --- | --- | --- | --- | --- |
| 5% | 17 | 16 | 33 | 418 | 96 |
| 10% | 33 | 32 | 65 | 434 | 112 |
| 20% | 65 | 64 | 129 | 466 | 144 |
| 40% | 129 | 128 | 257 | 530 | 208 |
| 60% | 193 | 192 | 385 | 594 | 272 |
| 80% | 257 | 256 | 513 | 658 | 336 |
